# Secondary Bracing Ligands Drive Heteroleptic Cuboctahedral Pd^II^_12_ Cage Formation

**DOI:** 10.1021/jacs.3c00661

**Published:** 2023-04-28

**Authors:** Carles Fuertes Espinosa, Tanya K. Ronson, Jonathan R. Nitschke

**Affiliations:** Yusuf Hamied Department of Chemistry, University of Cambridge, Lensfield Road, Cambridge CB2 1EW, United Kingdom

## Abstract

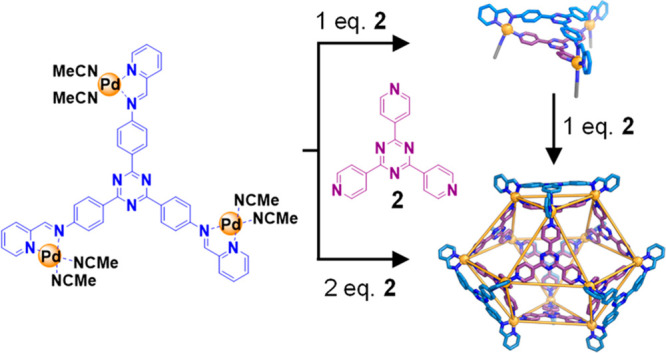

The structural complexity of self-assembled metal–organic capsules can be increased by incorporating two or more different ligands into a single discrete product. Such complexity can be useful, by enabling larger, less-symmetrical, or more guests to be bound. Here we describe a rational design strategy for the use of subcomponent self-assembly to selectively prepare a heteroleptic cage with a large cavity volume (2631 Å^3^) from simple, commercially available starting materials. Our strategy involves the initial isolation of a tris(iminopyridyl) Pd^II_3_^ complex **1**, which reacts with tris(pyridyl)triazine ligand **2** to form a heteroleptic sandwich-like architecture **3**. The tris(iminopyridyl) ligand within **3** serves as a “brace” to control the orientations of the labile coordination sites on the Pd^II^ centers. Self-assembly of **3** with additional **2** was thus directed to generate a large Pd^II_12_^ heteroleptic cuboctahedron host. This new cuboctahedron was observed to bind multiple polycyclic aromatic hydrocarbon guests simultaneously.

The use of coordination-driven self-assembly, where the coordination preferences of metal ions interact with the geometries of ligands to generate three-dimensional structures, provides a useful method for generating complex structures with minimal synthetic effort.^1^ Such architectures include helicates,^2^ grids,^3^ knots,^4^ and cages.^5^ Among the many different metal–organic assemblies that have been prepared, capsules are particularly attractive^6−9^ owing to their wide range of applications, including catalysis,^10^ chemical separation,^11^ stabilization of reactive species,^12^ and drug delivery.^13^

Cages of increasing complexity^14^ have been constructed using Pd^II^ and multitopic ligands,^15^ incorporating in some cases ligands that block two of the four available coordination sites.^16−18^ Many Pd^II^ coordination cages are homoleptic, containing one type of ligand.^19−22^ In order to increase the complexity and functionality of such systems, recent efforts have pursued the selective formation of heteroleptic assemblies,^23−27^ which contain more than one type of ligand.

Integrative self-sorting^28−32^ occurs when multiple ligands assemble together into a single discrete heteroleptic structure. Complementing this method, a stepwise strategy relies on the isolation of an initial coordination complex and its subsequent use as a building block for heteroleptic structures.^33,34^ As shown in the inset of Figure 1, the Fujita group reported^46^ a canonical example of this stepwise technique, involving the blockage of two *cis* coordination sites of square-planar Pd^II^ using ethylenediamine. The 90° angle between the remaining two Pd^II^ coordination sites thus defines the corner geometry of the product structure shown.

**Figure 1:**
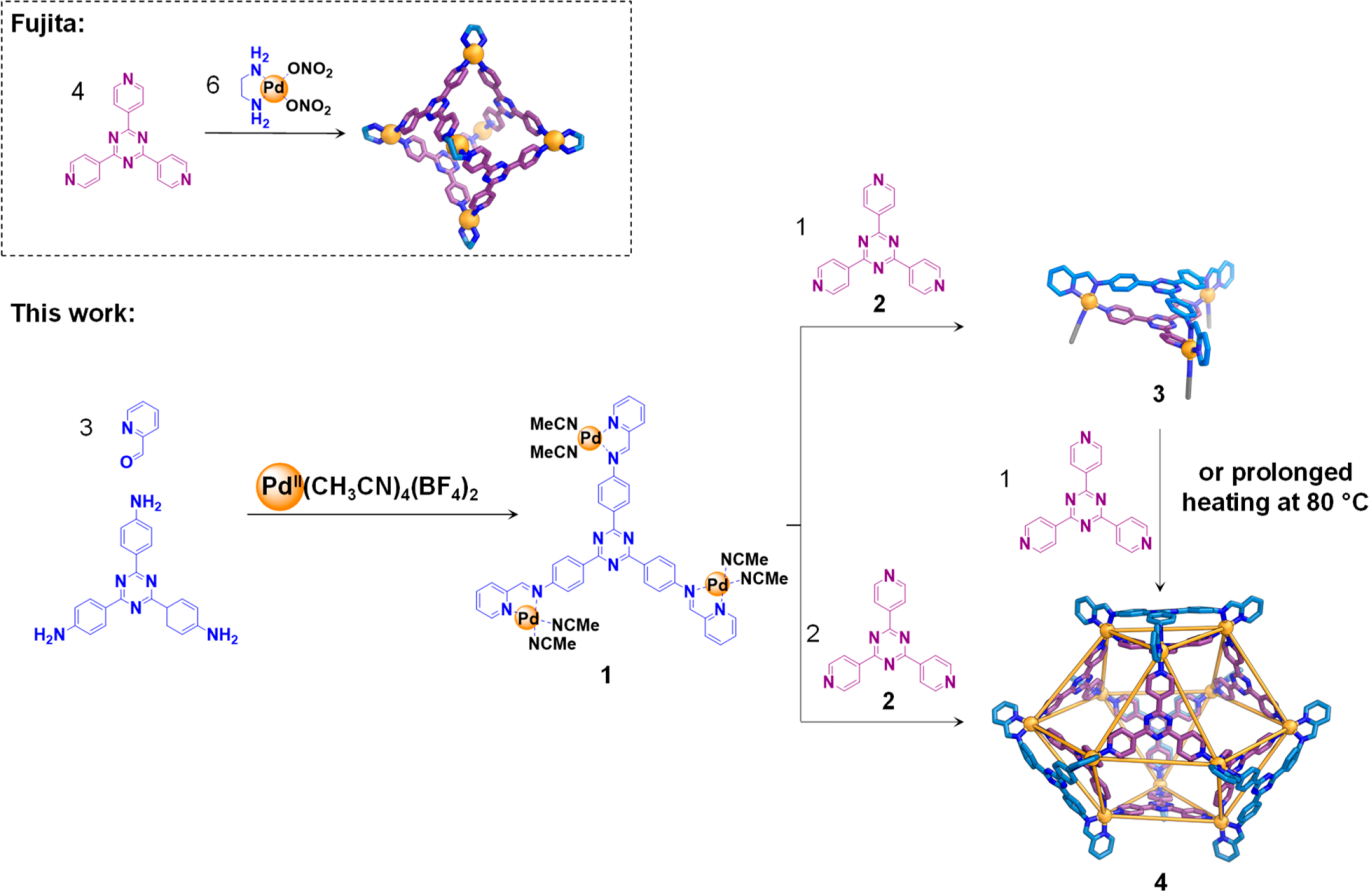
Synthesis of tris-Pd^II^ complex **1** and subsequent assembly of **3** and **4**. Inset, top: the self-assembly of Fujita’s original octahedral cage.^46^ Bottom: *cis*-ligated Pd^II^-complex **1**, and the subsequent self-assembly of 1 and **2** into sandwich-like structure **3** and cuboctahedron 4. Complexes **3** and 4 are shown as views from the crystal structures.

The present work builds upon and extends this strategy by controlling not only the coordination sphere of a single Pd^II^ center but also the relative orientations of coordination sites at three such centers at once within a single precursor subunit, enabling the synthesis of a large heteroleptic cage from simple subcomponents. Initial Pd^II^_3_ complex **1** (Figure 1) reacted with tris(pyridine) **2** to generate heteroleptic sandwich-like architecture **3**, whose acetonitrile ligands represent weak links^44^ that are readily displaced by bridging ligands. The secondary bracing ligand **2** within **3** generates tension within complex **3**, which orients the weak-link binding sites of its Pd^II^ centers. These three metal centers diverge in such a way as to prevent assembly into a small structure upon reaction with ligand **2**. Instead, 4 equiv of **2** serve to bridge 4 equiv of **3** within the framework of cuboctahedron **4**.

A mixture of 2,4,6-tris(4-aminophenyl)-1,3,5-triazine (1 equiv), 2-formylpyridine (3 equiv), and [Pd(CH_3_CN)_4_](BF_4_)_2_ (3 equiv) reacted in acetonitrile to form *cis*-ligated tri-Pd^II^ complex **1** (Figure 1), as confirmed by mass spectrometry and NMR spectroscopic analyses (Figures S1–S3). All characterization data are consistent with each Pd^II^ center in **1** being cis-coordinated by a pyridyl-imine ligand arm, with two coordination sites occupied by acetonitrile molecules to complete the square-planar coordination sphere.

The self-assembly of **1** with tritopic ligand 2,4,6-tris(4-pyridyl)-1,3,5-triazine **2** in acetonitrile yielded heteroleptic sandwich-like architecture **3** (Figure 1), as confirmed by mass spectrometry (Figure S4) and NMR (Figures S5–S10). Single crystals were grown by slow diffusion of ethyl acetate into an acetonitrile solution of **3**. X-ray analysis of a crystal using synchrotron radiation^45^ unambiguously revealed a heteroleptic structure in the solid state (Figures 1 and S11). The three Pd^II^ centers each coordinated an acetonitrile molecule to complete their square-planar coordination sphere. The Pd^II^ vertices are approximately equidistant from each other, with an average Pd···Pd distance of 12.85 Å. The close proximity (3.89 Å) between the centroids of the central triazine rings of the building blocks **1** and **2** within heteroleptic **3** precludes guest binding by eliminating any internal cavity (Figure S11).

We hypothesized that **3** had the potential to be used as a subunit for larger and more complex heteroleptic cages than the *pseudo*-octahedral cage originally reported by Fujita and co-workers (Figure 1, inset).^46^ The Fujita cage contains the same tritopic **2** ligands as **3**, but bridged by a ditopic (ethylenediamine)Pd^II^ subunit. This combination requires the formation of a structure with a Pd^II^_3n_**2**_2n_ composition, consistent with their observed (ethylenediamine) Pd^II^_6_L_4_ formulation. The tritopic heteroleptic configuration of **3** requires instead the formation of a **2**_n_**3**_n_ product. Strong mechanical coupling between the three Pd^II^ centers within **3** residues pushes the coordination vectors of their labile positions apart. The divergence of these coordination vectors thus disfavors the formation of smaller **2**_1_**3**_1_, **2**_2_**3**_2_ or **2**_3_**3**_3_ structures, in favor of the cuboctahedral **2**_4_**3**_4_ composition of **4**.

Cuboctahedron **4** could be prepared either through the reaction of **1** (1 equiv) and **2** (2 equiv), or by the reaction between equimolar amounts of **2** and **3**. Each of these assembly reactions produced **4** as the uniquely observed product (Figure 1), as confirmed by NMR spectroscopy (Figures S12–S18). All attempts to characterize **4** by high- and low-resolution mass spectrometry were unsuccessful, resulting in a series of peaks with +1 and +2 charge states, possibly corresponding to fragmentation.

The structure of **4** was unambiguously determined by X-ray crystallography at the Diamond Light Source synchrotron. Cuboctahedron **4** (Figure 2) has idealized *T*_d_ point symmetry. Its 12 Pd^II^ vertices are bridged by eight triangular faces, four of which are braced, corresponding to residues of heteroleptic assembly **3**; **4** contains six square windows that would allow passage of a sphere with a diameter as large as 12.7 Å. The residues of **3** within **4** retain their structure following incorporation into **4**; the bridging residues of **2** are held in the same orientation as the acetonitrile ligands in **3** (Figure S19). Cuboctahedron **4** surrounds a cavity volume of 2631 Å^3^ (Figure S46), calculated using Molovol.^47^

**Figure 2:**
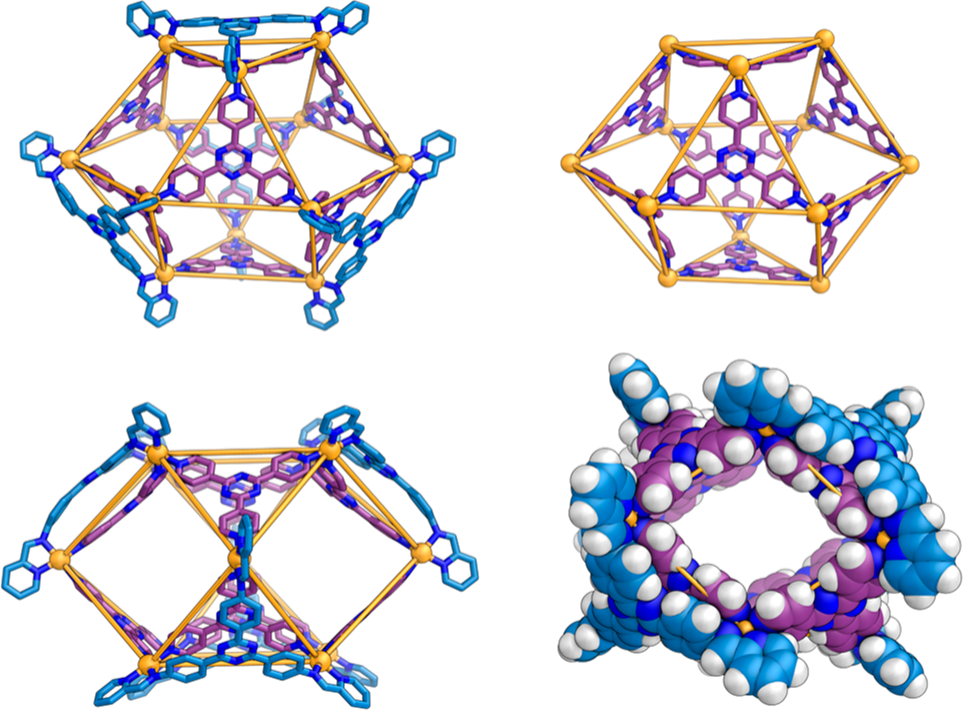
Views of the X-ray crystal structure of **4**, with the residues of cis-protected Pd^II^-complex **1** colored blue and ligand **2** colored purple. The top right shows the cuboctahedron core structure of **4** with the braces of **1** omitted; at lower right a space-filling view down the central cavity, with hydrogen atoms shown, which are omitted elsewhere. Anions and solvent are omitted for clarity.

The stepwise procedure shown in Figure 1 proved necessary to effect the synthesis of capsule **4**. When a one-pot preparation was attempted, in which all of the organic subcomponents incorporated into **4** were mixed with [Pd(CH_3_CN)_4_](BF_4_)_2_) in acetonitrile, a solid material was obtained that was not soluble in acetonitrile, chloroform, *N*,*N*-dimethylformamide, or dimethyl sulfoxide. ^1^H NMR analysis of the supernatant (Figure S20) revealed broad peaks that did not correspond to **3** or **4**. We infer that insoluble polymeric products formed and were kinetically trapped by precipitation under these conditions.

Either **3** or **4** can be obtained from the same building blocks, depending on stoichiometry and reaction conditions. When an acetonitrile solution of **3** was heated to 80 °C, ^1^H NMR monitoring revealed the complete conversion of **3** into **4** after 7 days (Figures 3 and S21). Lower temperatures resulted in incomplete conversion (Figures S49–S51). An insoluble material precipitated during this conversion. After removal of the insoluble precipitate generated during this transformation, an isolated yield of 91% of **4** was obtained, based upon the assumption that 8 equiv of **3** can transform into a maximum of 1 equiv of **4**.

**Figure 3:**
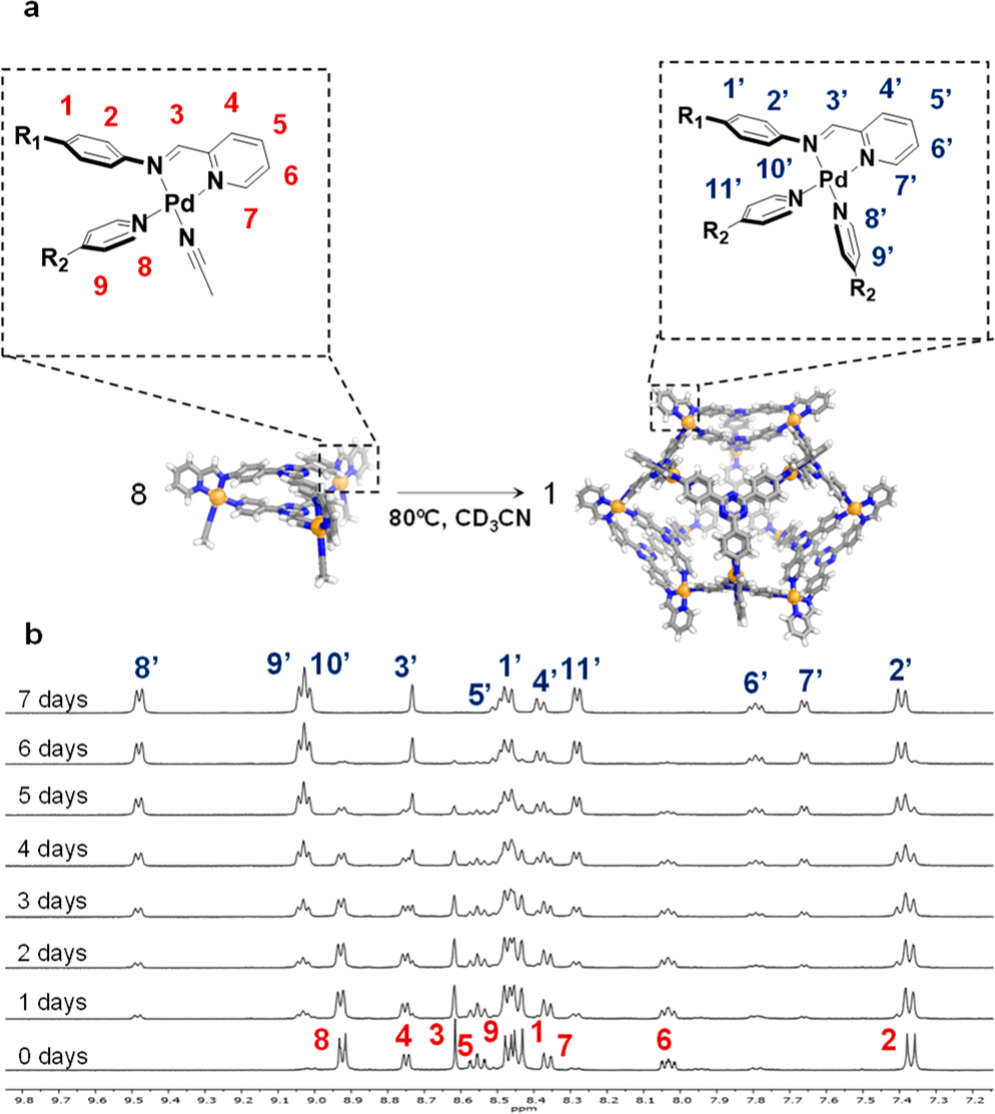
(a) Conversion of kinetic product **3** into the thermodynamic product **4**, with cut-outs showing the metal coordination sphere and ^1^H NMR assignments for **3** and **4**. (b) Partial ^1^H NMR spectra (CD_3_CN, 400 MHz, 298 K) monitoring the conversion of **3** (bottom) into **4** (top) at 80 °C.

This **3**-to-**4** transformation was inferred to proceed through a disassembly reassembly mechanism, in which **3** partially disassembled to release ligand **2** in solution, which reacted with the remaining **3** to form the cuboctahedral framework of **4**. We further observed that small amounts of **4** were always formed when the synthesis of **3** was carried out at 80 °C, even at short reaction times. We thus conclude **4** to be the thermodynamic product, even when less **2** was added than was required for its formation, with **3** being an isolable kinetic product.

A series of prospective guests for **4** were screened (Figure 4a), revealing the binding of PAHs (Figure 4), as indicated by ^1^H NMR (Figures S22–S36). In all cases, the signals of the guest underwent an upfield shift attributed to an inclusion-induced shielding effect, consistent with guest binding within the cavity of **4**. The pyridyl protons of the **2** residues paneling each triangular face of **4** also shifted downfield (Figures 4c and S22–S36), implicating these electron-deficient triazine panels in guest binding. When analogous control experiments using complex **1** or assembly **3** were carried out, no significant peak shifts were observed by ^1^H NMR, indicating that the cavity of **4** is crucial for the binding of PAHs. ^1^H DOSY NMR data for **4** indicated in all cases that the host–guest signals had similar diffusion coefficients as the signals of **4** (Figures S24, S27, S30, S33, and S37), consistent with host–guest complexation. We infer that donor–acceptor interactions drive binding of these guests. Conversely, larger PAHs, such as corannulene, coronene, or fullerenes, were not encapsulated within **4**.

**Figure 4:**
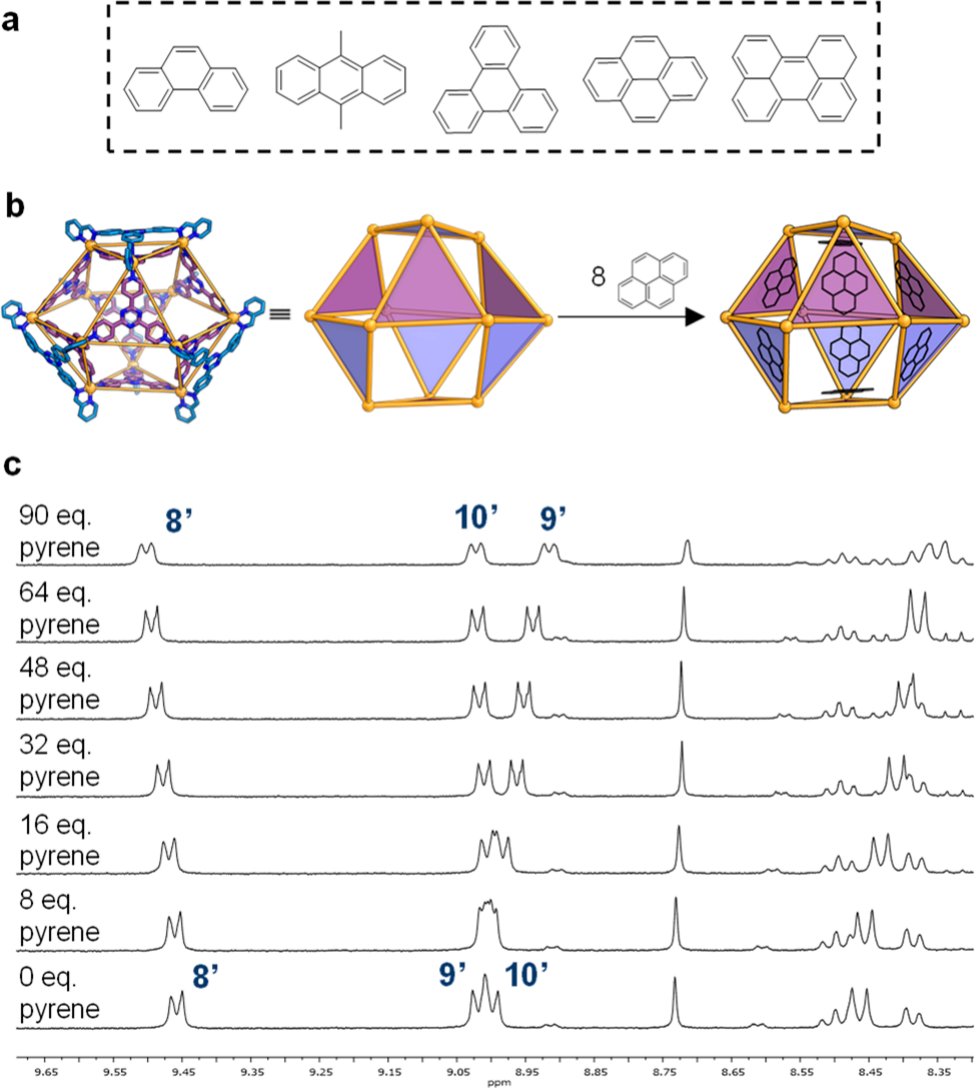
(a) Polyclic Aromatic Hydrocarbon guests for **4**. (b) Cartoon of the binding of eight molecules of pyrene within the cavity of **4**, showing interaction of each guest molecule with one of the eight triazine residues of **4**. (c) Partial ^1^H NMR spectra (400 MHz, CD_3_CN, 298 K) showing spectral changes during the progressive titration of pyrene in to a solution of **4**.

The 2631 Å^3^ cavity of **4** is large enough to bind multiple PAH guests. Integration of the ^1^H NMR spectra of these host–guest complexes indicates that each equivalent of **4** brings up to 8 equiv of the insoluble PAH into acetonitrile solution (Figures S22, S25, S28, S31, and S35), with guest binding occurring in all cases in fast exchange in the NMR time scale.

The low solubility of the PAHs in acetonitrile precluded quantification of their binding affinities, with the exception of pyrene. Titration of pyrene into **4** allowed the elucidation of host–guest stoichiometry and investigations of the cooperativity of multiple binding (Figures S40 and S41). The association constant *K*_a_ for pyrene was 1.17 (±0.1) × 10^2^ M^–1^. Job plot and mole ratio method analyses^48,49^ provided results consistent with the formation of a 1:8 host–guest complex between **4** and pyrene (Figures S38 and S39). Additionally, docking of pyrene into the crystal structure of **4** showed that eight pyrene units fitted within the cavity, occupying 59% of the total cavity volume (Figure S48). No evidence for either positive or negative cooperativity was revealed by a Hill plot (Figure S41).^50,51^ We infer this noncooperative binding to be a consequence of the independent interaction of each guest molecule with each of the eight triazine residues of **4**.

A series of competitive binding experiments were carried out to explore the relative binding affinity of **4** toward the different PAHs used, under the assumption that the ratio of host–guest complexes observed in solution reflects binding strength rather than simply guest solubility. An acetonitrile solution of **4** (1 equiv) was treated with an equimolar mixture of pyrene, perylene, triphenylene, phenanthrene, and 9,10-dimethyl anthracene (10 equiv each). After the suspension was stirred in acetonitrile for 2 h at 80 °C, the host–guest adducts were isolated and analyzed by ^1^H NMR (Figure S42), indicating the preferential formation of pyrene ⊂ 4 (66%) and perylene ⊂ 4 (33%), while the signals of the other guests present in the mixture were not observed. An analogous experiment was carried out excluding pyrene from the equimolar guest mixture. The ^1^H NMR spectrum of the resulting mixture indicated the selective encapsulation of perylene (75%) and triphenylene (25%) (Figure S43). These results suggested binding affinities of **4** toward the different PAHs studied in the order pyrene > perylene > triphenylene ≈ phenanthrene ≈ 9,10-dimethyl anthracene (Figures S42–44).

The straightforward preparation of cuboctahedron **4** thus represents a novel approach to the design of larger, heteroleptic hosts, capable of binding multiple guests. Our strategy of extending subcomponent self-assembly to the combination of multiple metal centers into a single preorganized subunit may prove useful in generating further large, heteroleptic structures, potentially capable of binding more complex, lower-symmetry guest species, such as biomolecules, along with collections of guests. Different secondary ligands may also enable the incorporation of **3** residues into lower-symmetry heteroleptic cages, capable of binding lower-symmetry guests.

## Supporting Information Available

Experimental procedures; NMR characterizations; mass spectrometry data; volume calculations; X-ray crystallographic data CCDC 2175722 for **3** CCDC 2175723 for **4** (PDF)
