# Supplementary material for: Secondary Bracing Ligands Drive Heteroleptic Cuboctahedral PdII12 Cage Formation
Source: J Am Chem Soc. 2023 Apr 28;145(18):9965–9. doi: 10.1021/jacs.3c00661 (PMC10176475; doi:10.1021/jacs.3c00661)
Supplement: Supplementary file 1 — ja3c00661_si_001.pdf [file ja3c00661_si_001.pdf]

Supporting Information

Secondary Bracing Ligands Drive Heteroleptic Cuboctahedral Pd^{II}_{12} Cage Formation

Carles Fuertes Espinosa, Tanya K. Ronson, and Jonathan R. Nitschke*

Yusuf Hamied Department of Chemistry, University of Cambridge, Lensfield Road, Cambridge, United Kingdom, CB2 1EW

jrn34@cam.ac.uk

Table of Contents

1. Supplemental Experimental Procedures 3

1.1 General Information 3

1.2 Synthesis and characterisation of 1 4

1.3 Synthesis and characterisation of 3 7

1.4 Self-assembly and characterisation of 4 12

1.5 Conversion of a solution of 3 to 4 17

2. Host-guest binding studies of 4 18

2.1 Preparation and characterization of the host-guest complexes 19

2.2 Determination of the association ratio and binding constant for the pyrene ⊂4 host-guest complex 28

2.3 Competitive encapsulation experiments 30

3 X-ray crystallography 32

4 Volume calculations 35

5 Molecular Modelling 36

6 References 36

1. Supplemental Experimental Procedures

1.1 General Information

Unless otherwise stated all starting materials were sourced from commercial suppliers and used without further purification. Self-assembly reactions were conducted in d_3-MeCN.

NMR spectra were recorded using the following NMR spectrometers: Bruker 400 MHz Avance III HD Smart Probe (routine ^{1}H NMR, ^{1}H DOSY, host-guest ^{1}H NMR titration and ^{1}H NMR to monitor the conversion of **3** to **4**), 500 MHz DCH Cryoprobe (High resolution ^{1}H, ^{13}C and 2D NMR experiments). Chemical shifts (δ) were reported in parts per million (ppm) for ^{1}H and ^{13}C spectra. Chemical shifts were referenced using the residual CD_3CN solvent signal (^{1}H = 1.94 ppm, ^{13}C = 118.26 ppm). Signal multiplicity in ^{1}H NMR spectra was described using the following abbreviations: singlet (s), doublet (d), triplet (t), doublet of doublets (dd), triplet of doublets (td), doublet of doublet of doublets (ddd), multiplet (m), broad (br) and apparent (app.).

^{1}H DOSY NMR experiments were conducted on a Bruker 400 MHz Avance III HD Smart Probe spectrometer. Maximum gradient strength was 5.35 G/cm A. The standard Bruker pulse program, ledbpgp2s,[3] employing a stimulated echo and longitudinal eddy-current delay (LED) using bipolar gradient pulses for diffusion using 2 spoil gradients, was utilized. A gradient ramp of 10% to 80% was used. d20 was set to 0.1 s and p30 was optimised for each species.

Low resolution electrospray ionisation mass spectra (ESI–LRMS) were recorded on a Micromass Quattro LC instrument (cone voltage 10 or 20 eV; capillary voltage 3.8 kV; desolvation temperature 313 K; source block temperature 313 K), infused from a Harvard Syringe Pump at a rate of 10 µL per minute.

1.2 Synthesis and characterisation of 1

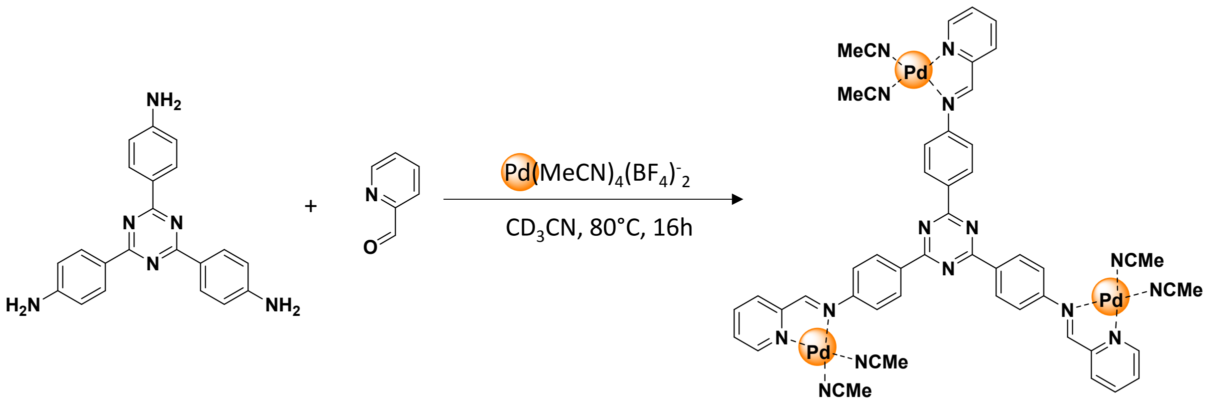

1

Scheme S1. Synthesis of cis-protected Pd^{II}-complex **1**

To a round bottom flask, 4,4',4''-(1,3,5-triazine-2,4,6-triyl)trianiline (10 mg, 6 µmol, 1.0 equiv.), 2-formylpyridine (8.5 µL, 18 µmol, 3 equiv.) and CD_3CN (2.5 mL) were added. To this suspension, $[Pd(CH_3CN)_4](BF_4)_2$ (44mg, 18 µmol, 3 equiv.) was added and the resulting orange solution was stirred at 80 °C for 16 hours. The reaction mixture was allowed to cool to room temperature, filtered through a glass fibre plug and Et_2O (*ca.* 10 mL) was added. The orange precipitate was collected using centrifugation and washed with Et_2O (3 x 15 mL). The solid was suspended in a mixture of MeCN and DCM (1:3 MeCN:DCM, 12 mL), excess lithium bis(trifluoromethane)sulfonimidate (25 mg, 50 µmol, 24 equiv.) was added and the mixture was stirred vigorously at room temperature for 16 hours. The resulting orange solution was filtered through a glass fibre plug, concentrated *in vacuo* and Et_2O (*ca.* 10 mL) was added. [**1**$(NTf_2)_6$] was collected using centrifugation and washed with Et_2O (3 x 15 mL). Finally [**1**$(NTf_2)_6$] was obtained as an orange solid (25.9 mg, 87%).

^{1}H NMR (500 MHz, CD_3CN, 298 K) δ 9.04 (d, *J* = 8.8 Hz, 6H), 8.61 (d, *J* = 8.2 Hz, 3H), 8.50 (t, *J* = 7.9, 3H), 8.48 (s, 3H), 8.32 (d, J = 7.3 Hz, 3H), 7.96 (t, J = 7.8 Hz, 3H), 7.82 (d, *J* = 6.8 Hz, 6H).

ESI–LRMS [**1**$(NTf_2)_6$] = $C_{39}H_{27}N_9Pd_3$ $(C_2F_6NO_4S_2)_6$) *m/z* = 197.7 [**1**$(NTf_2)_0]^{6+}$ (calc. 197.8), 294.7 [**1**$(NTf_2)_1]^{5+}$ (calc. 294.8), 440.3 [**1**$(NTf_2)_2]^{4+}$ (calc. 440.4).

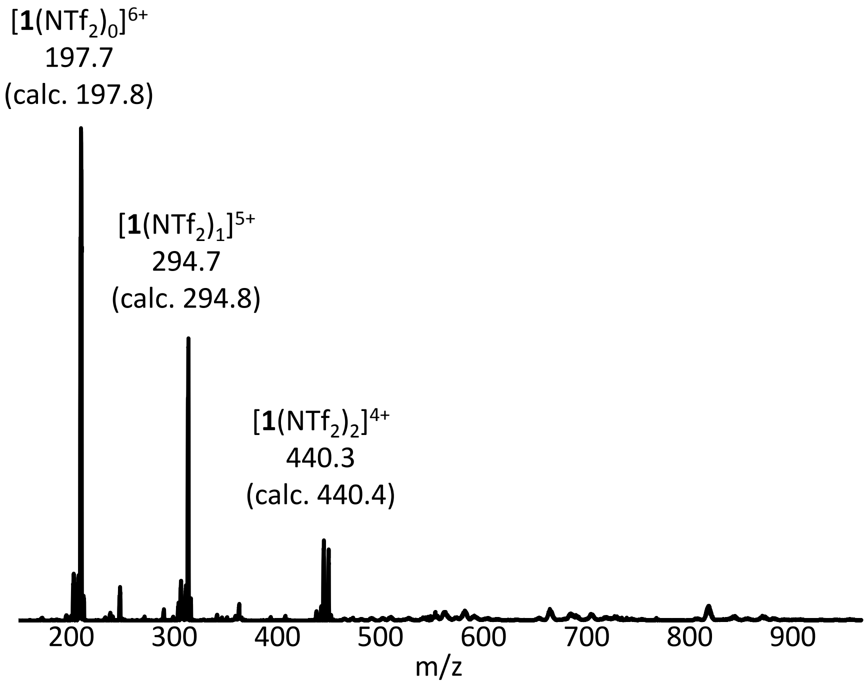

Figure S1. Low resolution ESI-mass spectrum for **1**.

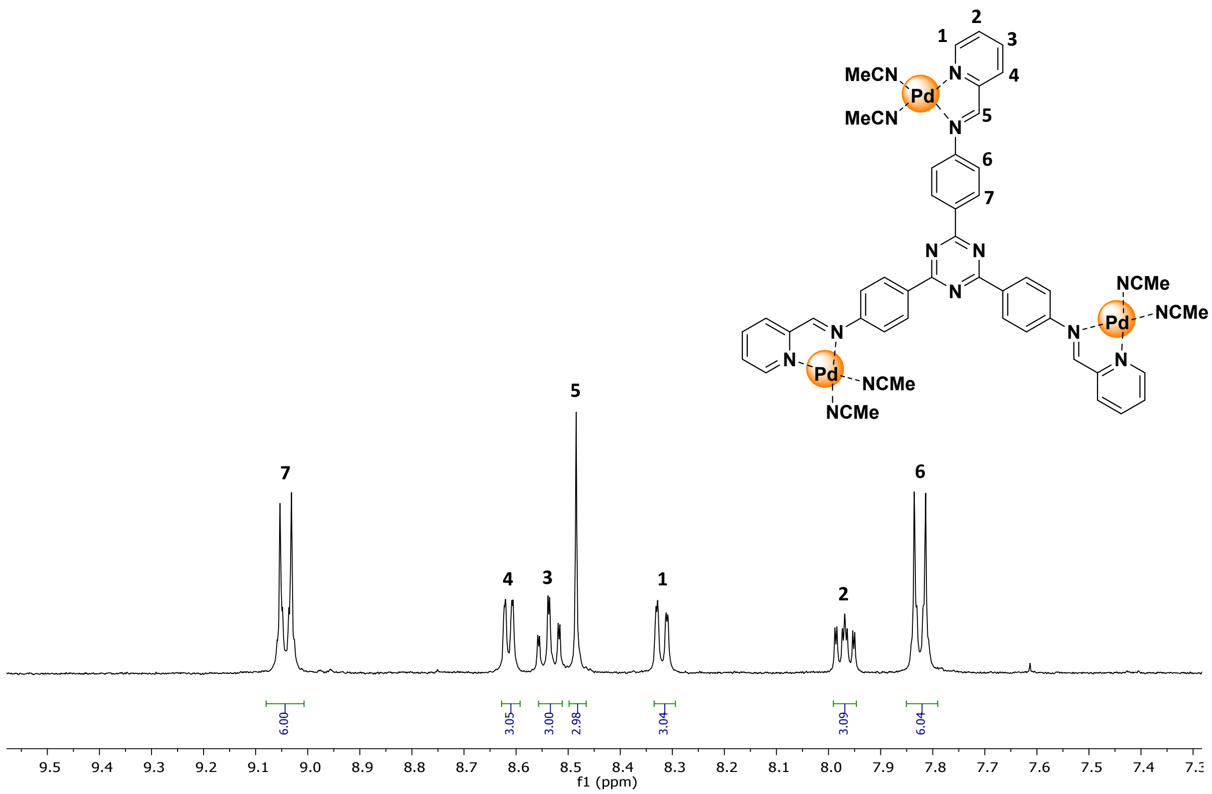

Figure S2. Aromatic region of the ^1H NMR spectrum (500 MHz, CD_3CN, 298 K) of **1**.

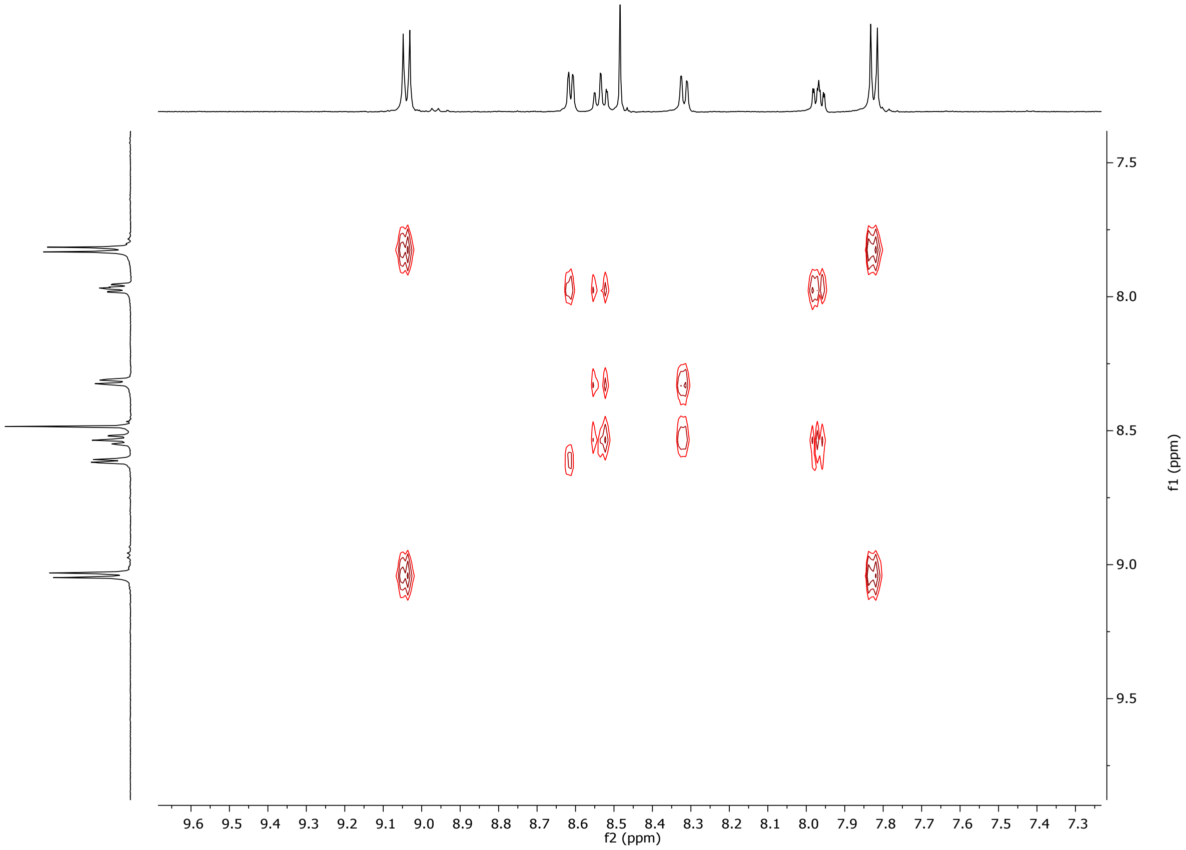

Figure S3. Aromatic region of the ^1H-^1H DQF-COSY spectrum (500 MHz, CD_3CN, 298 K) of **1**.

1.3 Synthesis and characterisation of 3

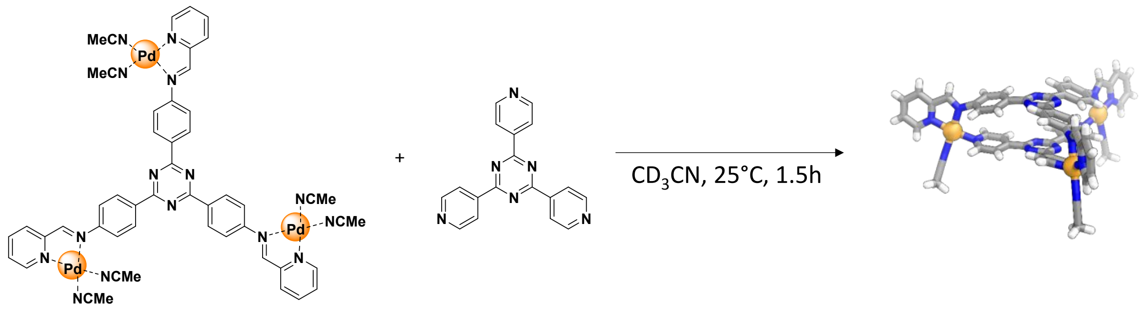

1

3

Scheme S2. Self-assembly of **3**

To a round bottom flask, Pd^{II}-complex **1** (6 mg, 3 µmol, 1.0 equiv.), 2,4,6-tris(4-pyridyl)-1,3,5- (0.78 mg, 3 µmol, 1 equiv.) and CD_3CN (0.5 mL) were added. The yellow/orange suspension was stirred at 25 °C for 1.5 hours. The resulting solution was concentrated using a stream of N_2 and Et_2O (ca. 15 mL) was added. The yellow/orange precipitate was collected using centrifugation and washed with Et_2O (3 x 15 mL). **3** was obtained as a yellow/orange solid (6.72 mg, 3 µmol, quant.).

^{1}**H NMR** (500 MHz, CD_3CN, 298 K) δ 8.95 (d, J = 8.9 Hz, 6H), 8.77 (d, *J* = 8.05 Hz, 3H), 8.65 (s, 3H), 8.57 (t, J = 8.20 Hz, 3H), 8.49 (d, *J* = 8.0 Hz, 6H), 8.47 (d, J = 8.2 Hz, 6H), 8.39 (d, J = 7.9 Hz, 3H), 8.05 (t, J = 8.07 Hz, 3H), 7.39 (d, *J* = 7.4 Hz, 6H).

^{13}**C NMR** (126 MHz, CD_3CN, 298 K) δ 176.2, 171.0, 170.3, 155.6, 154.7, 154.0, 148.7, 145.7, 144.6, 136.3, 132.3, 131.9, 131.3, 126.3, 124.5, 122.12, 120.8 (q, *J* = 321 Hz, NTf_2).

ESI–LRMS ([**3**$(NTf_2)_6$] = [$(C_{57}H_{39}N_{15}Pd_3)(C_2F_6NO_4S_2)_6$] *m/z* = 236.8 [**3**$(NTf_2)_0]^{6+}$ (calc. 236.9), 342.1 [**3**$(NTf_2)_1]^{5+}$ (calc. 342.2), 498.7 [**3**$(NTf_2)_2]^{4+}$ (calc. 498.8).

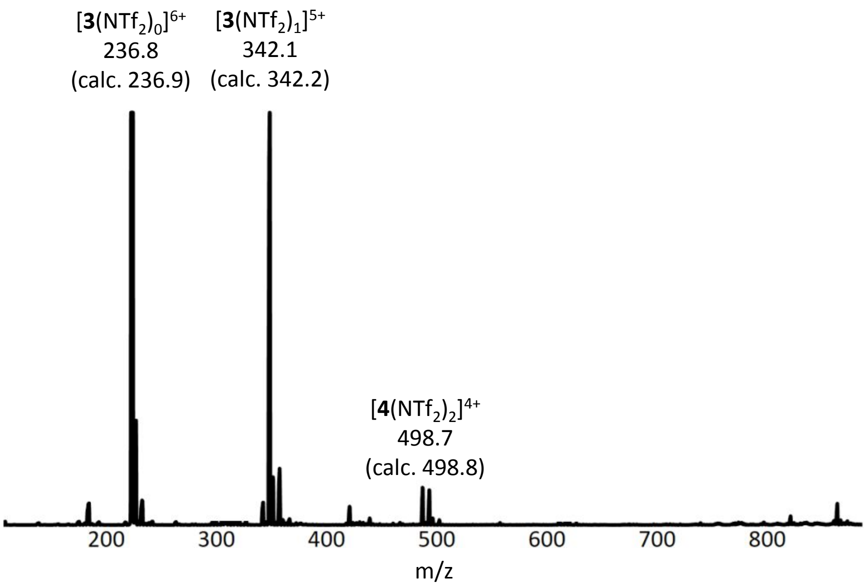

Figure S4. Low resolution ESI-mass spectrum for **3**.

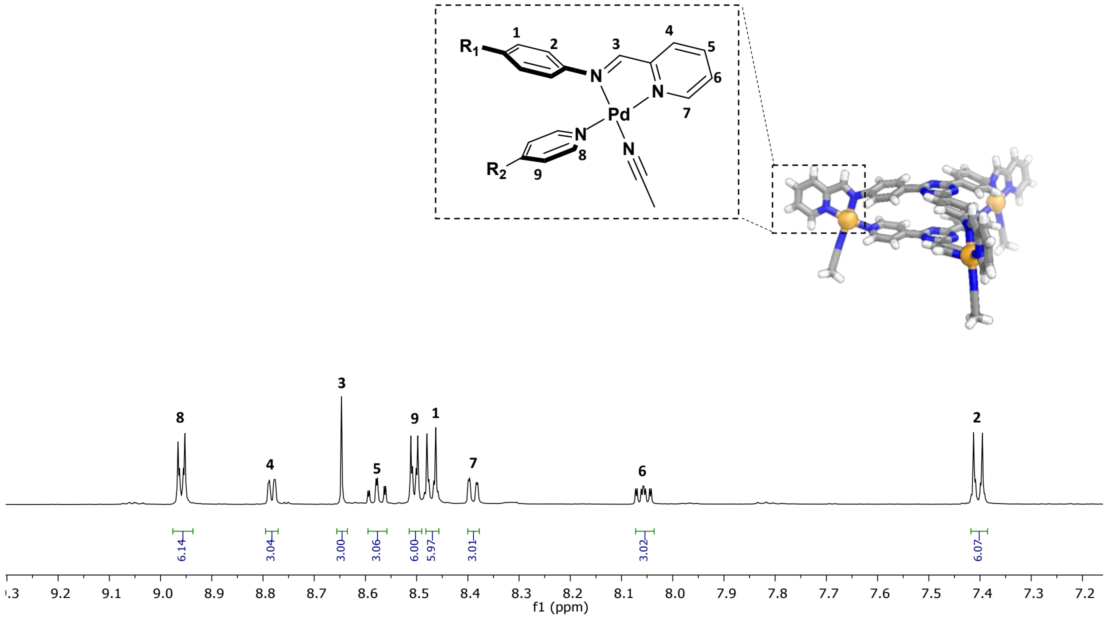

Figure S5. Aromatic region of the ^1H NMR spectrum (500 MHz, CD_3CN, 298 K) of **3**.

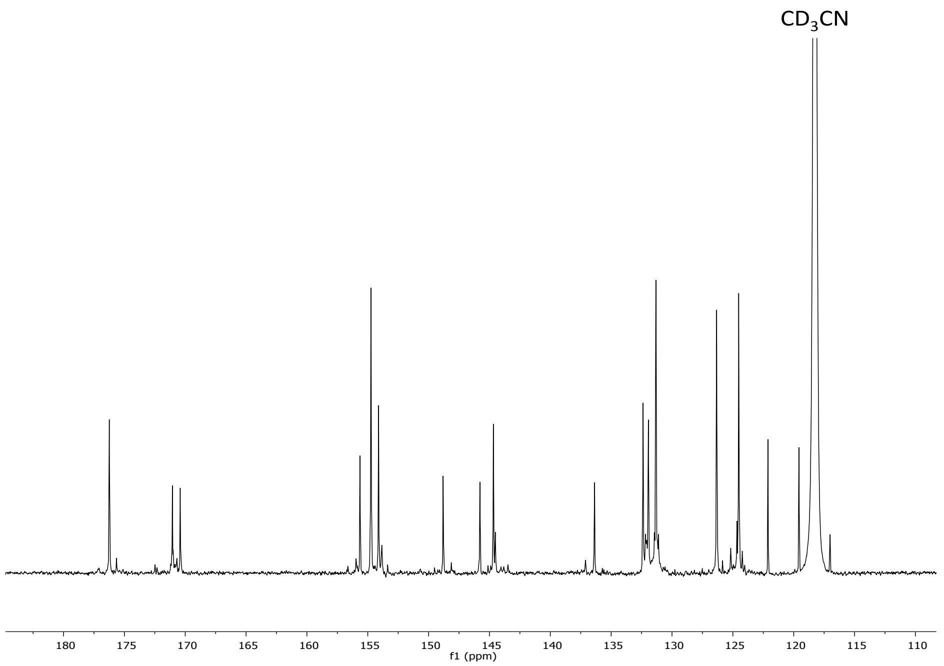

Figure S6. Aromatic region of the ^{13}C NMR spectrum (126 MHz, CD_3CN, 298 K) of **3**.

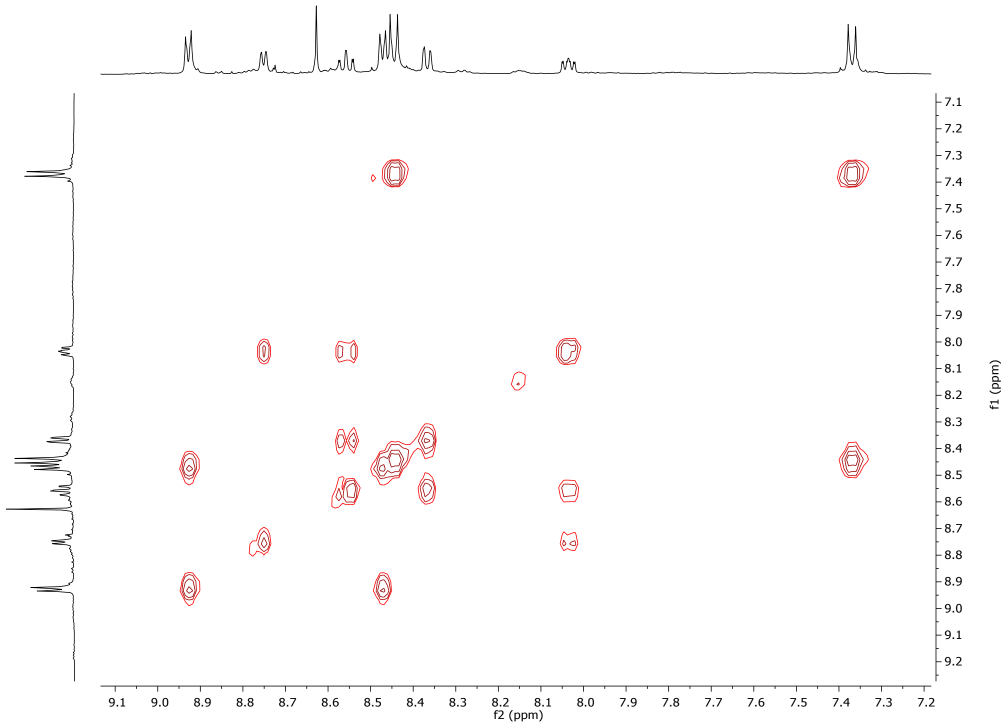

Figure S7. Aromatic region of the ^1H-^1H DQF-COSY spectrum (500 MHz, CD_3CN, 298 K) of **3**.

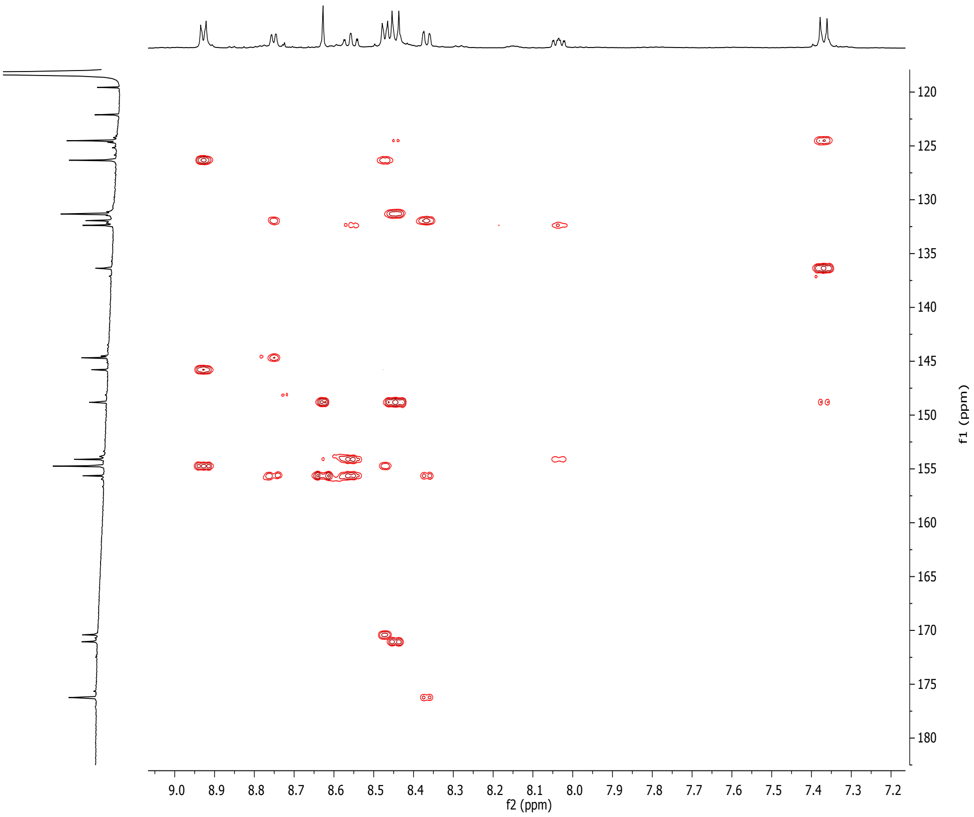

Figure S8. Aromatic region of the ^{1}H-^{13}C HMBC spectrum (500 MHz, CD_3CN, 298 K) of **3**.

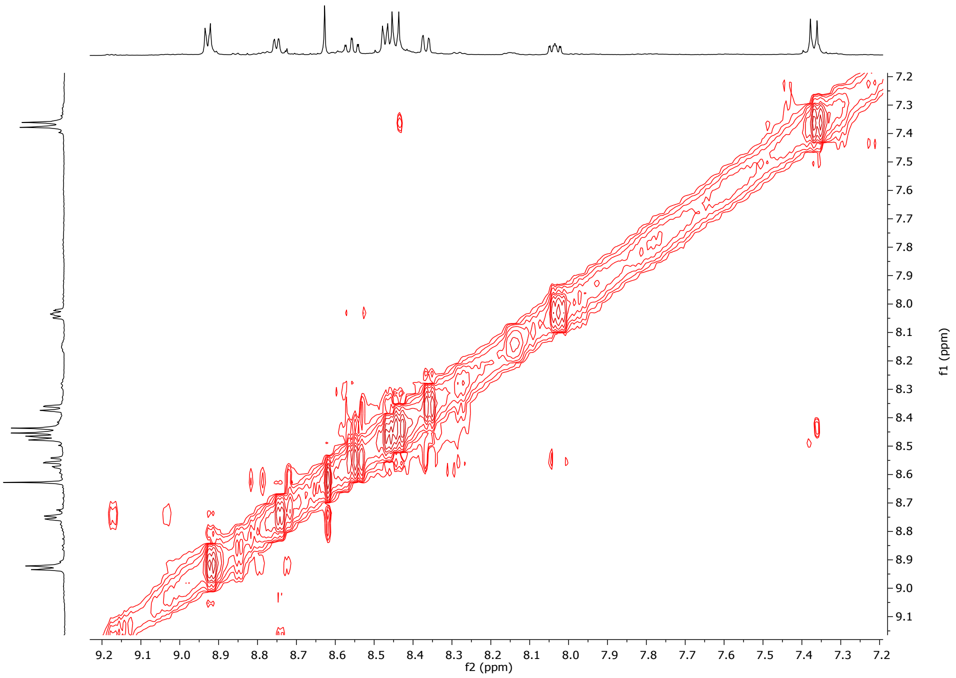

Figure S9. Aromatic region of ^{1}H-^{1}H NOESY spectrum (500 MHz, CD_3CN, 298 K) of **3**.

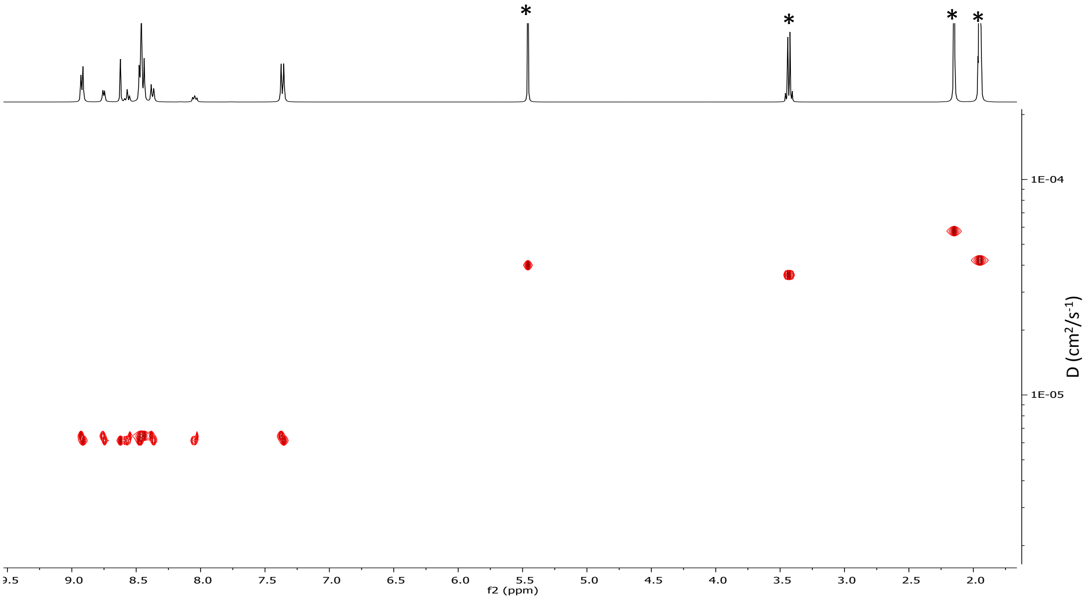

Figure S10. ^{1}H DOSY spectrum (400 MHz, CD_3CN, 298 K) of **3**. The diffusion coefficient for this species in CD_3CN was measured to be 6.82 x 10^{-6} cm^2 s^{-1}. * indicate signals for protons of residual solvents.

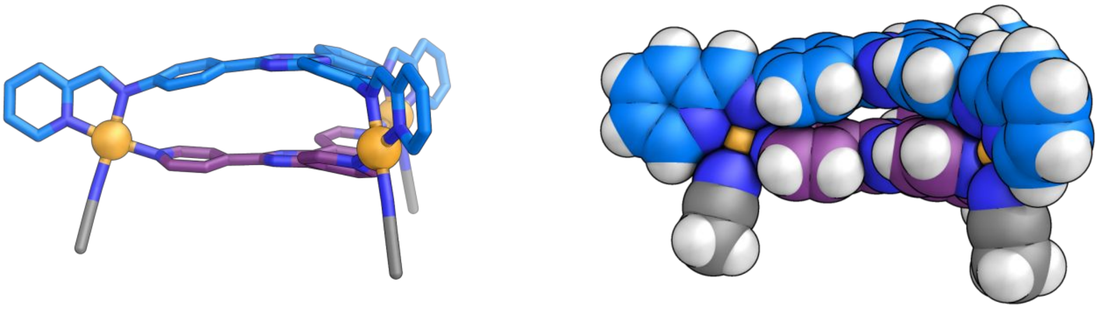

Figure S11. View of crystal structure of **3** (left) and space-filling mode (right)

1.4 Self-assembly and characterisation of 4

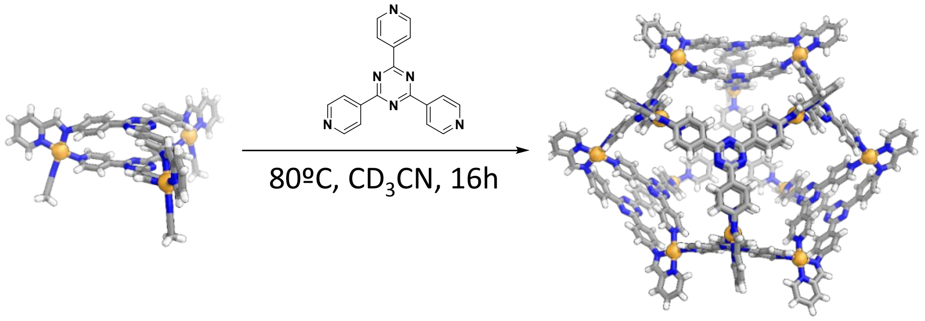

3 **4**

Scheme S3. Self-assembly of **4**

To a round bottom flask, assembly **3** (6.7 mg, 3 µmol, 1.0 equiv.) and 2,4,6-tris(4-pyridyl)-1,3,5- (0.78 mg, 3 µmol, 1 equiv.) and CD_3CN (0.5 mL) were added. The yellow suspension was stirred at 80 °C for 16 hours. The resulting solution was concentrated using a stream of N_2 and Et_2O (ca. 15 mL) was added. The yellow precipitate was collected using centrifugation and washed with Et_2O (3 x 15 mL). **3** was obtained as a yellow solid (4.9 mg, 0.75 µmol, quant.).

^{1}H NMR (500 MHz, CD_3CN, 298 K) δ 9.5 (d, J = 9.1 Hz, 24H), 9.06 (d, *J* = 8.8 Hz, 24H), 9.04 (d, J = 8.8 Hz, 24H), 8.76 (s, 12H), 8.51 (t, *J* = 8.3 Hz, 12H), 8.49 (d, J = 8.6 Hz, 24H), 8.39 (d, J = 8.4 Hz, 12H), 8.31 (d, J = 7.9 Hz, 24H), 7.82 (t, *J* = 7.5 Hz, 12H), 7.68 (d, *J* = 7.7 Hz, 12H), 6.8 (d, *J* = 7.9 Hz 24H).

^{13}C NMR (126 MHz, CD_3CN, 298 K) δ 173.4, 170.0, 169.7, 169.2, 154.9, 153.4, 152.9, 151.7, 148.1, 146.0, 144.4, 143.3, 135.1, 131.3, 130.7, 130.3, 126.5, 125.2, 123.7, 123.3, 121.1, 120.8 (q, *J* = 321 Hz, NTf_2).

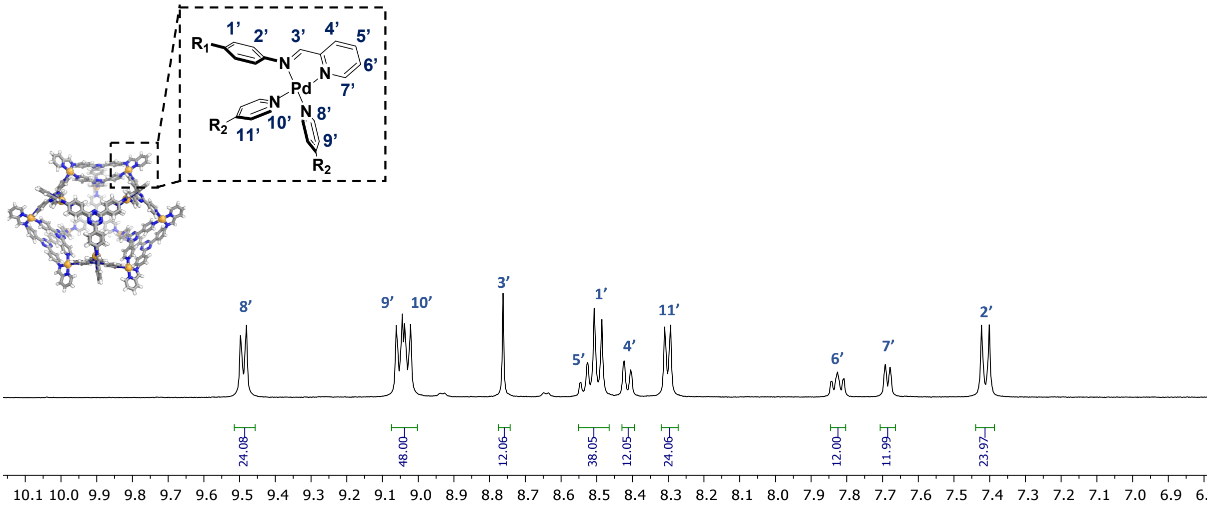

Figure S12. Aromatic region of the ^{1}H NMR spectrum (500 MHz, CD_3CN, 298 K) of **4**.

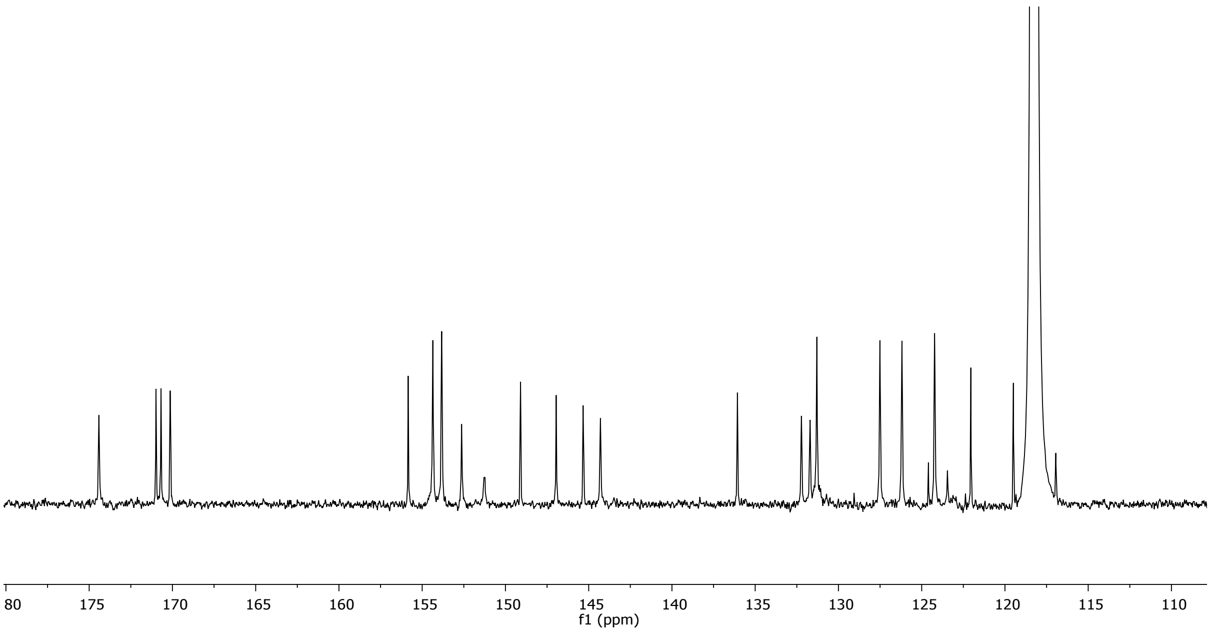

Figure S13. Aromatic region of the ^{13}C NMR spectrum (126 MHz, CD_3CN, 298 K) of **4**.

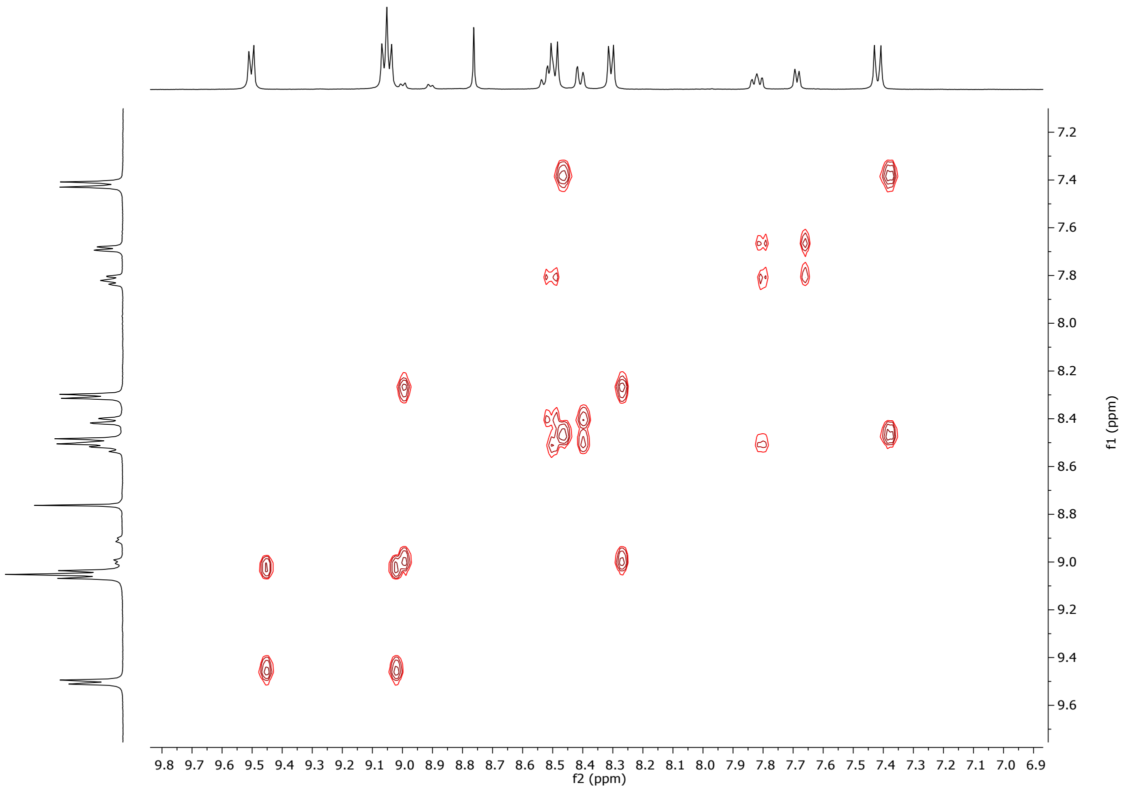

Figure S14. Aromatic region of the ^{1}H-^{1}H DQF-COSY spectrum (500 MHz, CD_3CN, 298 K) of **4**.

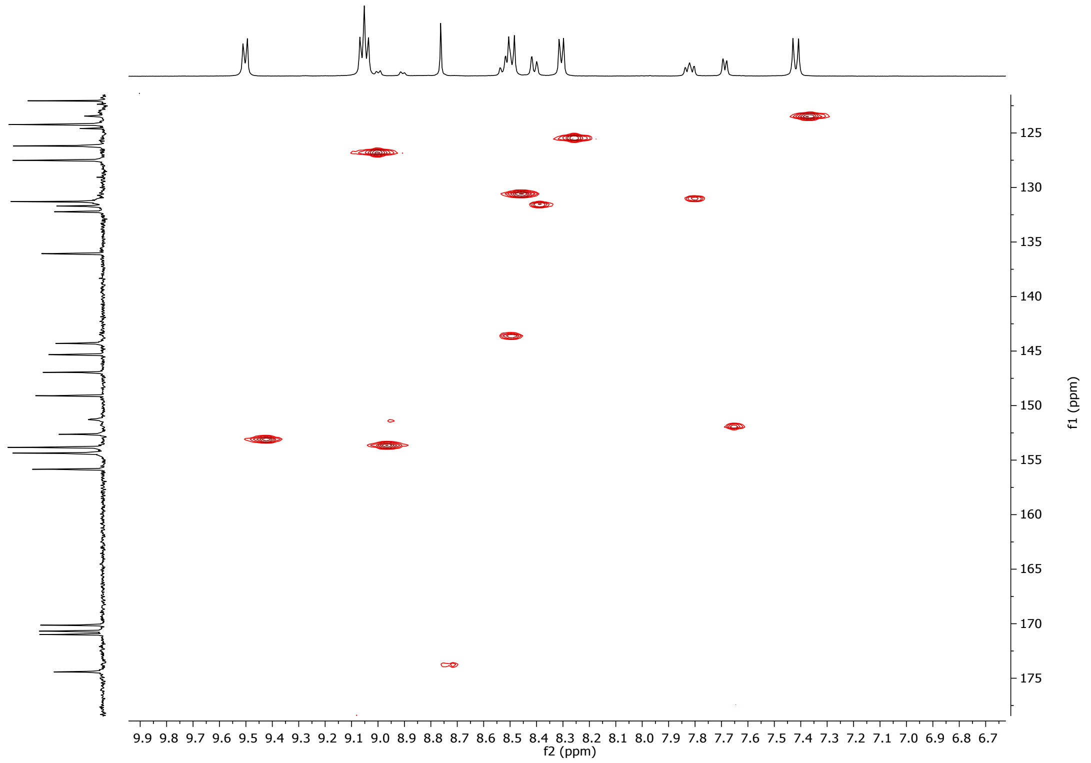

Figure S15. Aromatic region of the ^{1}H-^{13}C HSQC spectrum (500 MHz, CD_3CN, 298 K) of **4**

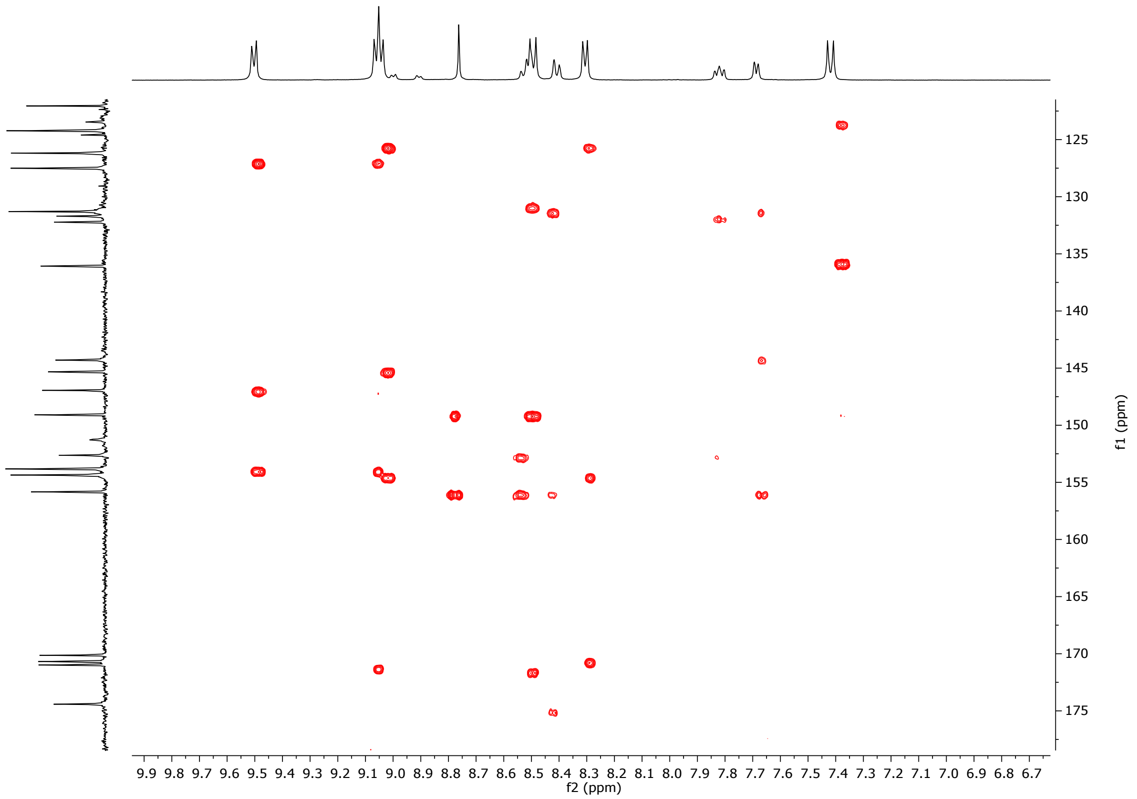

Figure S16. Aromatic region of the ^{1}H-^{13}C HMBC spectrum (500 MHz, CD_3CN, 298 K) of **4**.

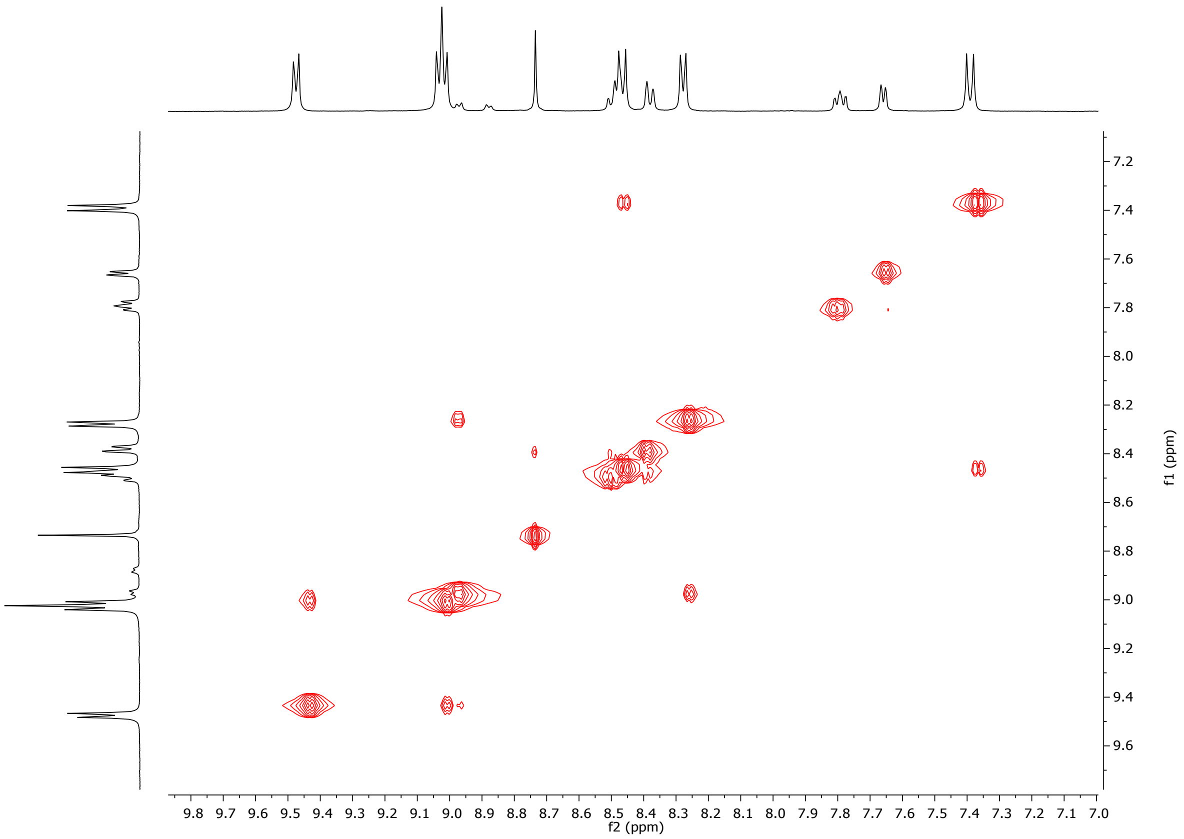

Figure S17. Aromatic region of ^{1}H-^{1}H NOESY spectrum (500 MHz, CD_3CN, 298 K) of **4**.

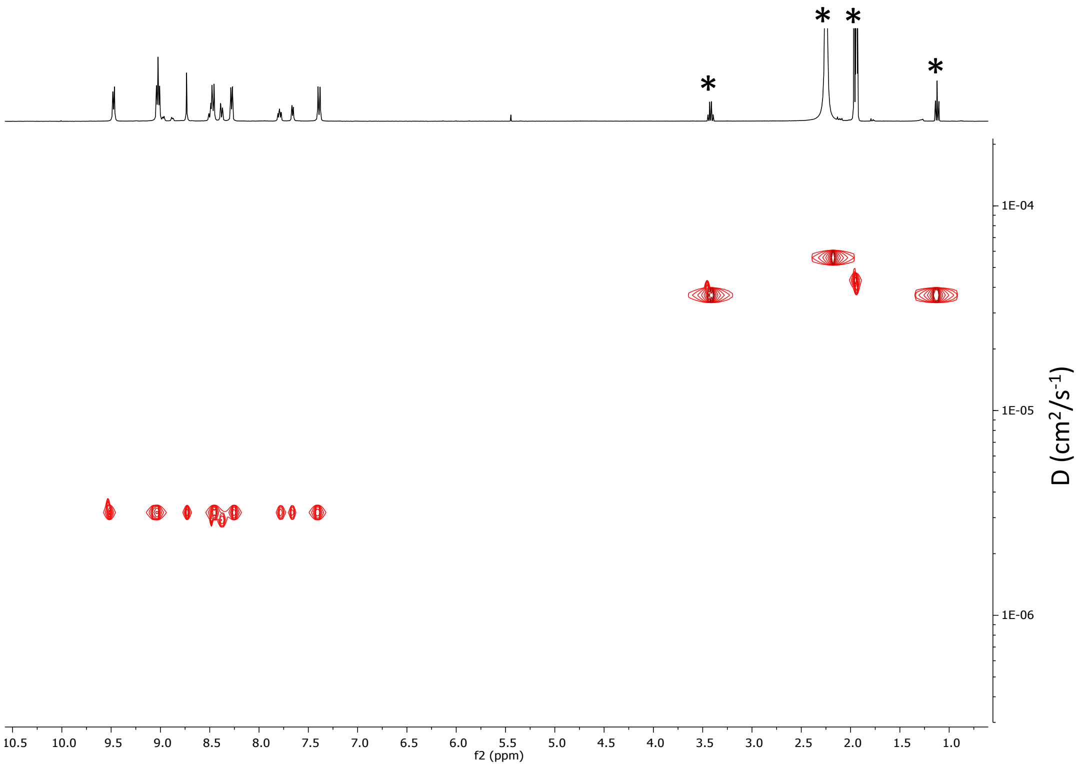

Figure S18. ^{1}H DOSY spectrum (400 MHz, CD_3CN, 298 K) of **4**. The diffusion coefficient for this species in CD_3CN was measured to be 3.42 x 10^{-6} cm^2 s^{-1}. * indicate signals for protons of residual solvents.

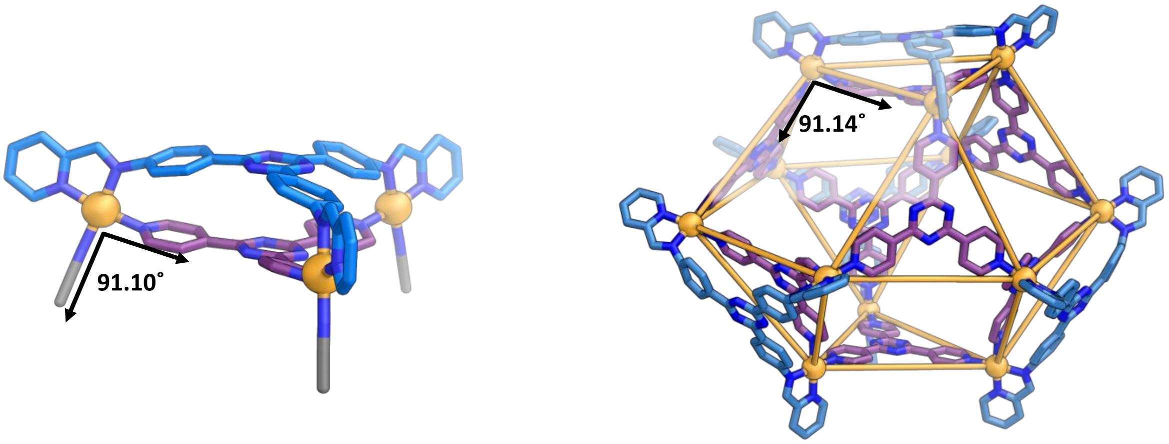

Figure S19. View of crystal structure of **3** (left) and **4** (right).

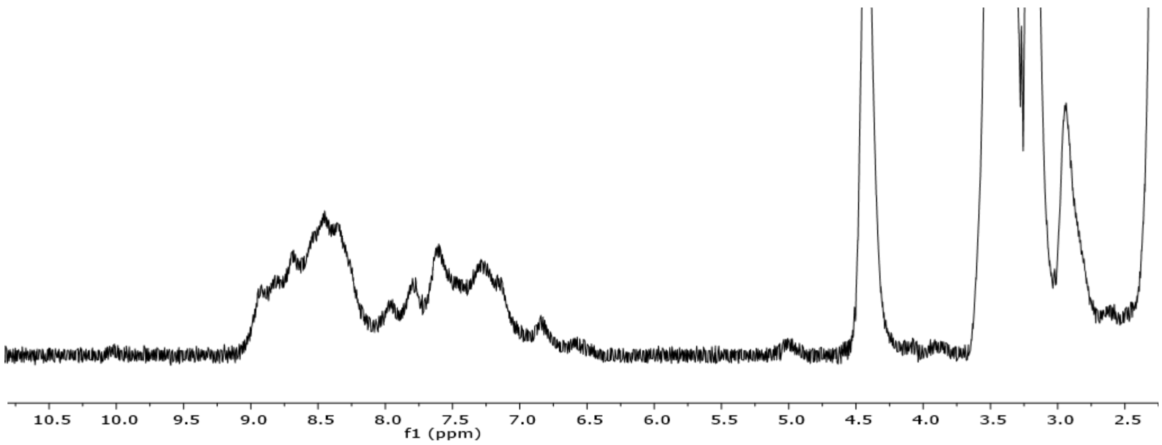

Figure S20. ^1H NMR spectrum (500 MHz, CD_3CN, 298 K) of the reaction mixture from the attempted one pot synthesis of **4**. The signals for **4** were not observed, indicating that the two step procedure was necessary for the formation of **4**.

1.5 Conversion of a solution of 3 to 4

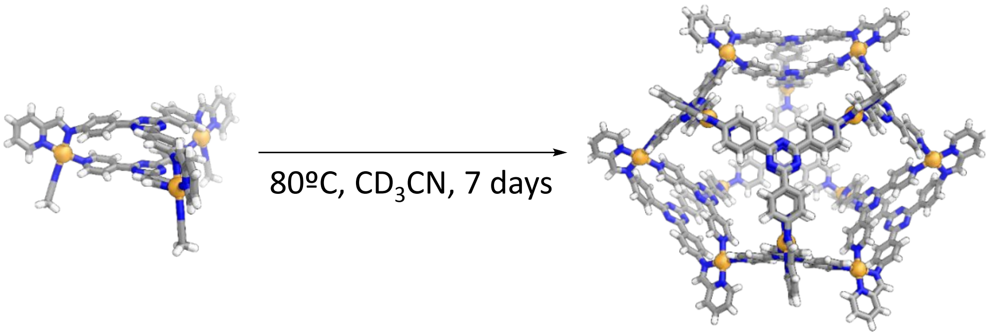

3 **4**

Scheme S4. Conversion of **3** to **4**

A 0.5 mL solution of **3** (200 µM) in CD_3CN was prepared, using 1,3,5-trimethoxybenzene as an internal standard for the calibration of concentration. The sample was heated to 80 °C in a NMR tube. Over 7 days, every 24 hours the sample was filtered to remove the solid generated and analyzed by ^1H NMR. At the end of the experiment, the resulting solution was concentrated using a stream of N_2 and Et_2O (ca. 15 mL) was added. The yellow precipitate was collected by centrifugation, washed with Et_2O (3 x 15 mL) and dried under vacuum. Product **4** was obtained as a yellow solid (4.2 mg, 91% yield).

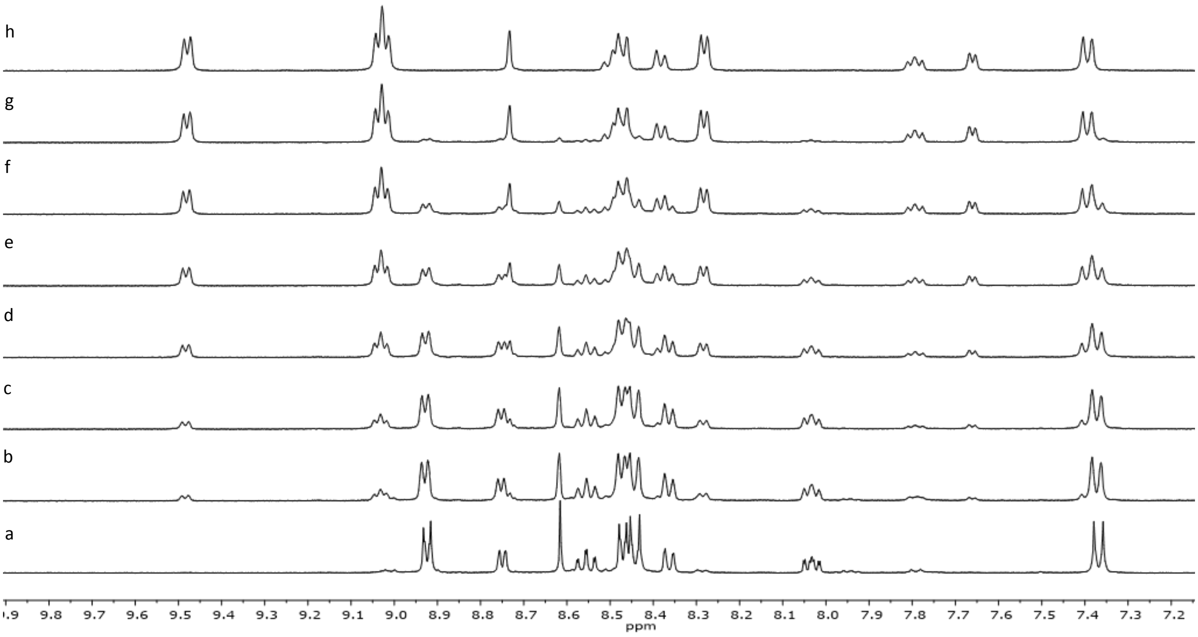

Figure S21. ^1H NMR spectra (500 MHz, CD_3CN, 298 K) of a solution of **3** in CD_3CN with an initial concentration of approx. 200 µM. 0 d, and then after heating at 80 °C for (b) 1 d (c) 2 d (d) 3 d (e) 4 d (f) 5 d (g) 6 d (h) 7 d .The final spectrum matches that of freshly prepared **4**. The significant upfield shift of some of the peripheral protons of **4** when compared with their corresponding analogues in **3**, it is inferred to be a consequence of the ring current effect from the nearby pyridine rings from the triazine residues.

2. Host-guest binding studies of 4

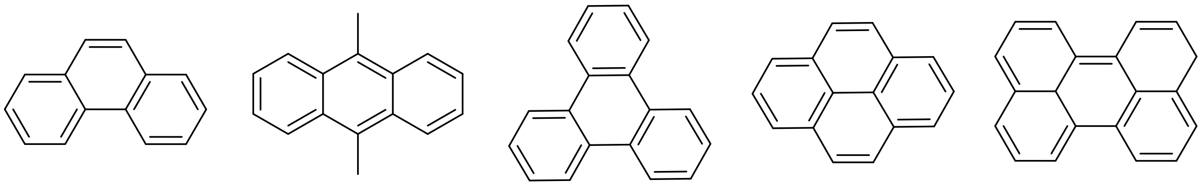

Scheme S4. PAHs used for the host-guest binding studies

A series of structurally similar PAH molecules, including phenanthrene, 9,10-dimethyl anthracene, triphenylene, pyrene and perylene were investigated as potential guests for cage **4.** The binding studies were carried out in CD_3CN and were investigated by monitoring the changes of the ^1H NMR peaks of both the cage and the guests. It was found all the PAH guests interacted with the cage in a fast-exchange process. Titration experiments also showed that the binding between pyrene and cage **4** was fast to reach equilibrium, taking around 1 min at 25 °C after mixing. The low solubility of the other PAH molecules in acetonitrile prevented quantification of their association constants through ^1H NMR titration. The binding constant for pyrene was determined.

2.1 Preparation and characterization of the host-guest complexes

A 0.5 mL solution of **4** (approx. 160 µM) in CD_3CN was prepared, using 1,3,5-trimethoxybenzene as an internal standard for calibration of concentration. To this solution, 10 equiv. of the corresponding PAH was added. The obtained suspension was stirred at 80ºC for 2 hours, filtered and Et_2O (ca. 15 mL) was added. The yellow precipitate was collected using centrifugation.

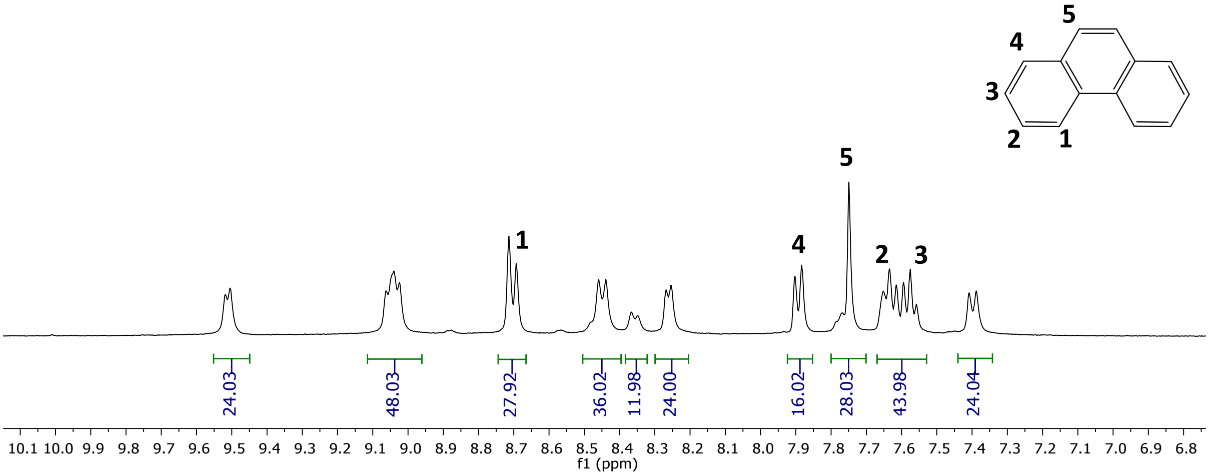

Figure S22. Aromatic region of the ^1H NMR spectrum (500 MHz, CD_3CN, 298 K) of the prepared phenanthrene ⊂ **4** host-guest complex.

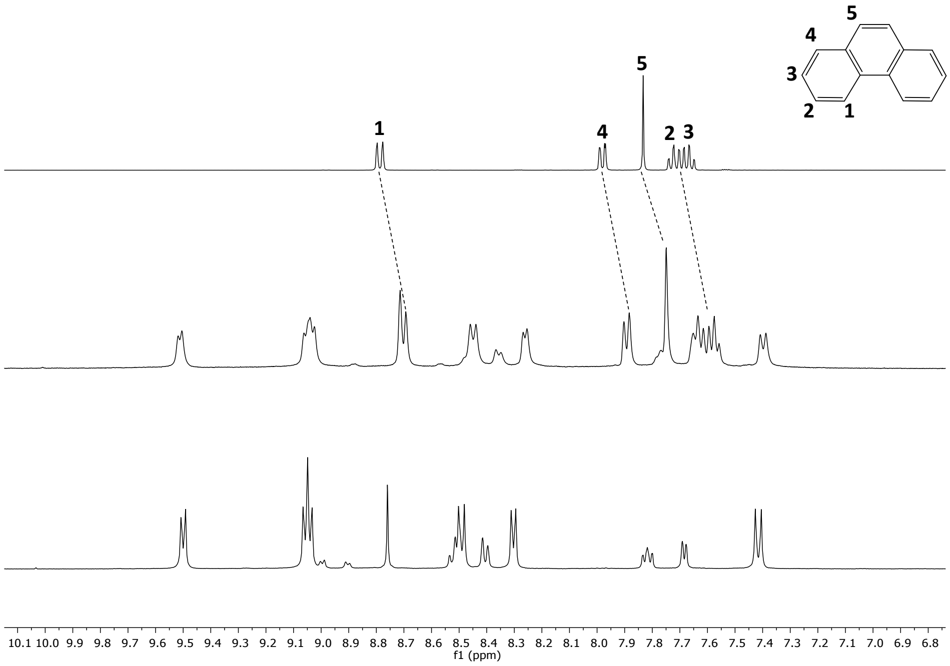

Figure S23. Aromatic region of the ^{1}H NMR spectra (500 MHz, CD_3CN, 298 K) of **4** (bottom), the prepared phenanthrene ⊂ **4** host-guest complex (middle) and phenanthrene (top)

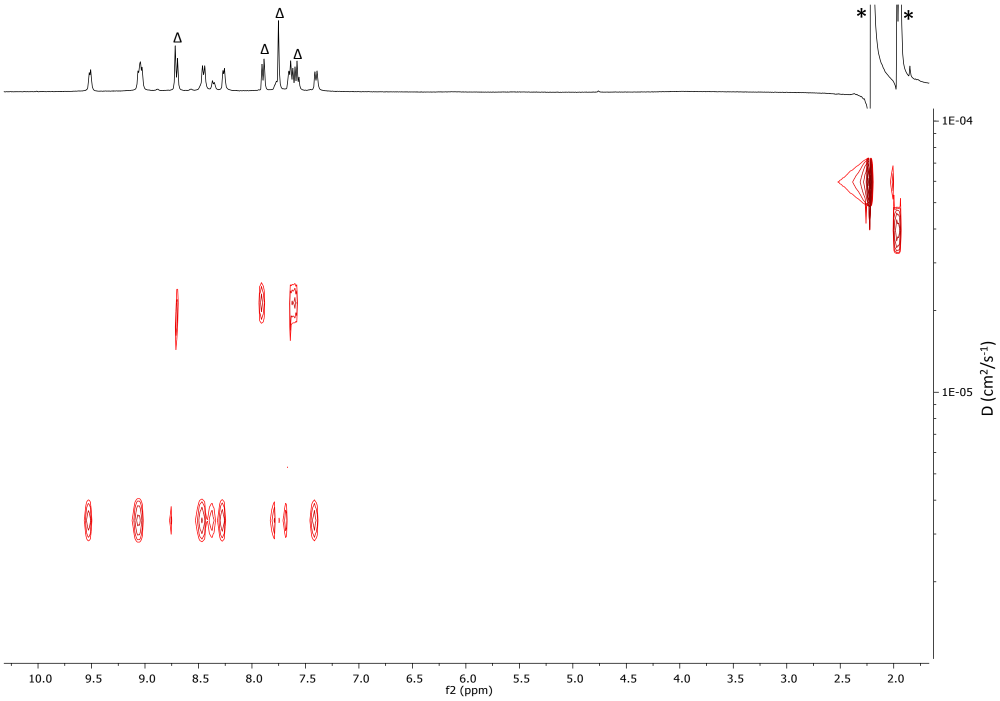

Figure S24. ^{1}H DOSY spectrum (400 MHz, CD_3CN, 298 K) of the phenanthrene ⊂ **4** host-guest complex. The diffusion coefficients for the host and the guest species in CD_3CN were measured to be 3.40 x 10^{-6} $cm^2\,s^{-1}$ and 2.55 x 10^{-5} $cm^2\,s^{-1}$ respectively. Δ indicate signals for protons of the guest and * indicate signals for protons of residual solvents.

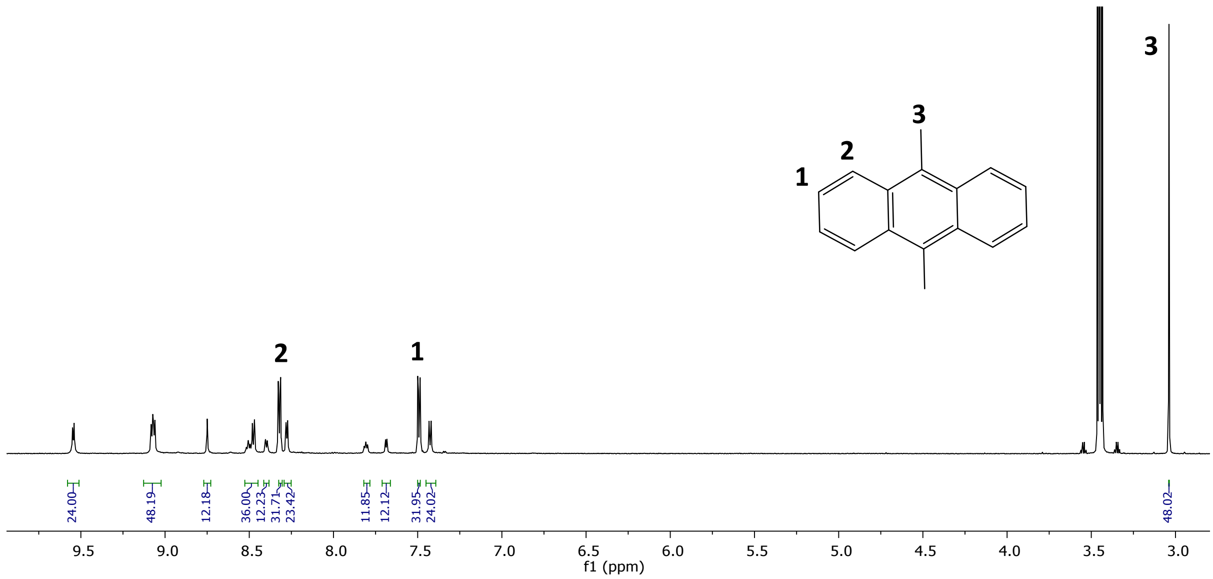

Figure S25. ^{1}H NMR spectrum (500 MHz, CD_3CN, 298 K) of the prepared 9,10-dimethyl anthracene ⊂ **4** host-guest complex.

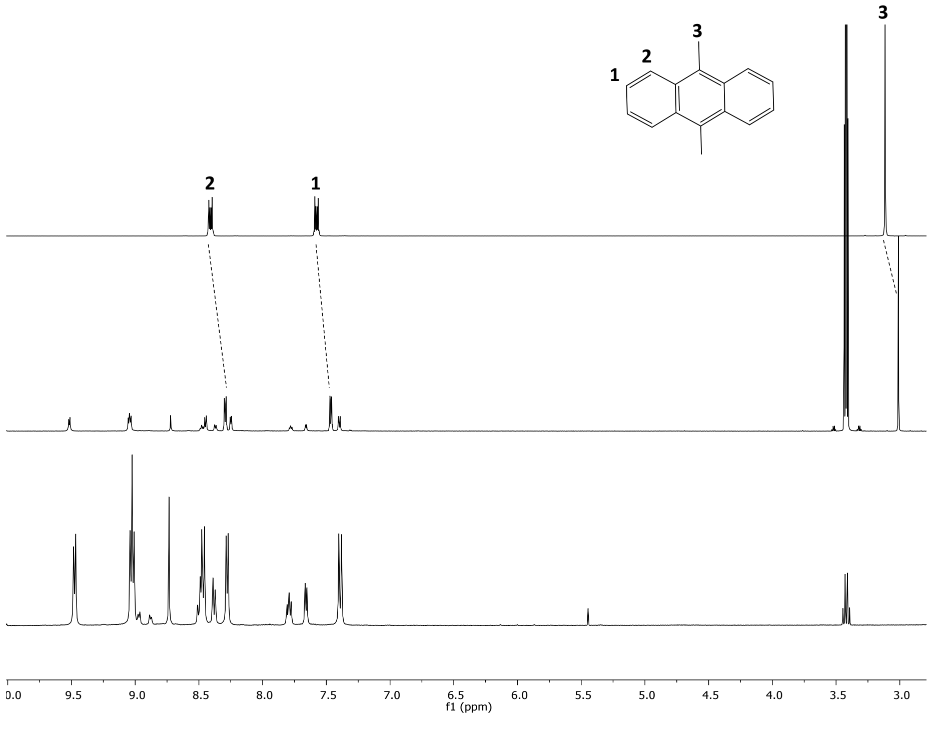

Figure S26. Aromatic region of the ^{1}H NMR spectra (500 MHz, CD_3CN, 298 K) of **4** (bottom), the prepared 9,10-dimethyl anthracene ⊂ **4** host-guest complex (middle) and anthracene (top)

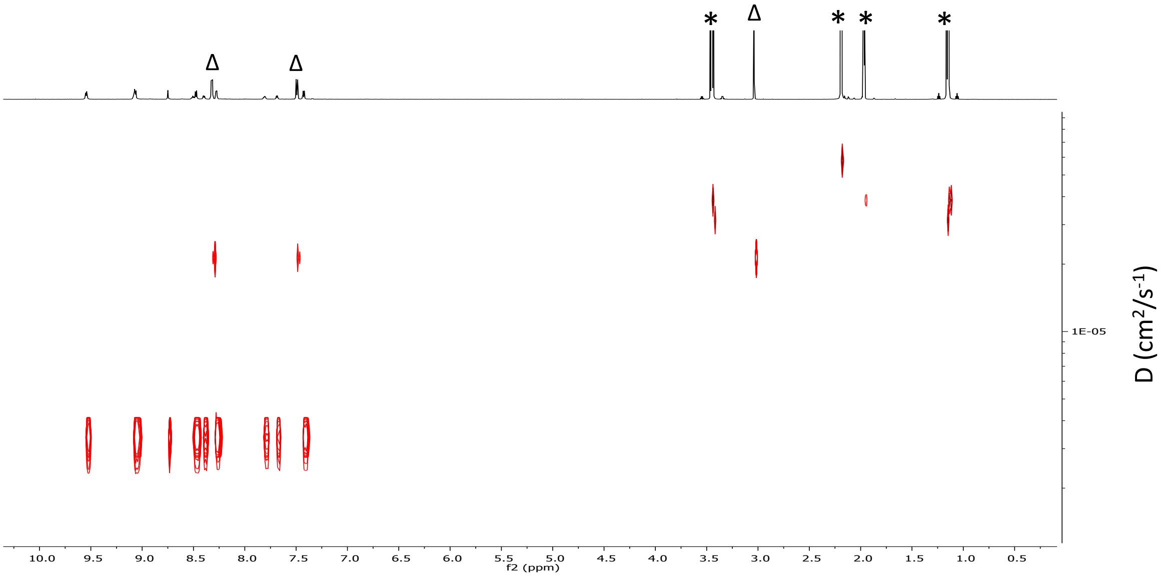

Figure S27. ^{1}H DOSY spectrum (400 MHz, CD_3CN, 298 K) of 9,10-dimethyl anthracene ⊂ **4** host-guest complex. The diffusion coefficients for the host and the guest species in CD_3CN were measured to be 3.38 x 10^{-6} cm^2 s^{-1} and 2.64 x 10^{-5} cm^2 s^{-1} respectively. Δ indicate signals for protons of guest and * indicate signals for protons of residual solvents.

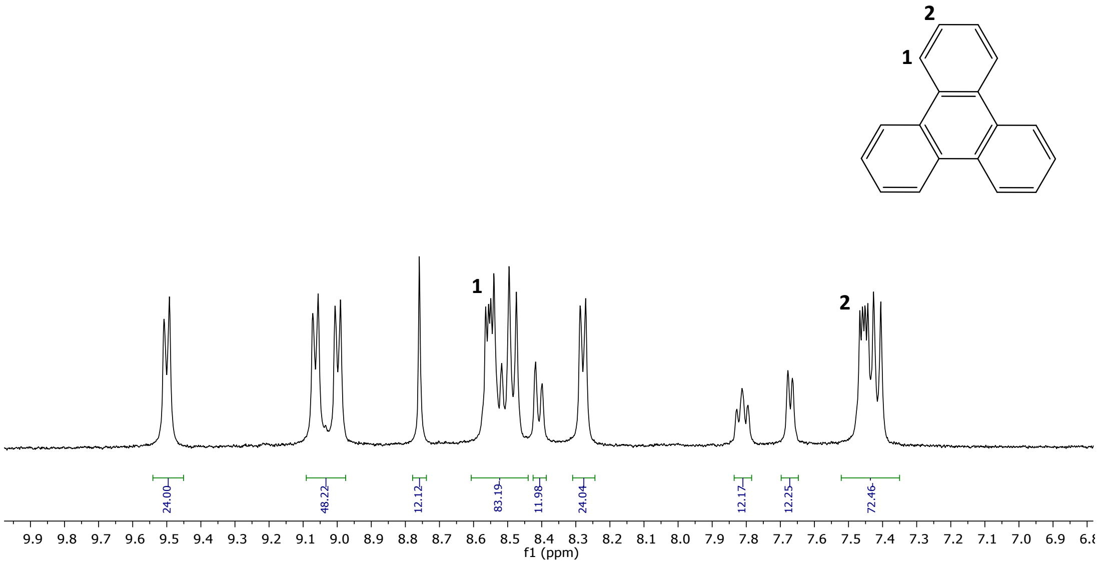

Figure S28. Aromatic region of the ^{1}H NMR spectrum (500 MHz, CD_3CN, 298 K) of the prepared triphenylene ⊂ **4** host-guest complex

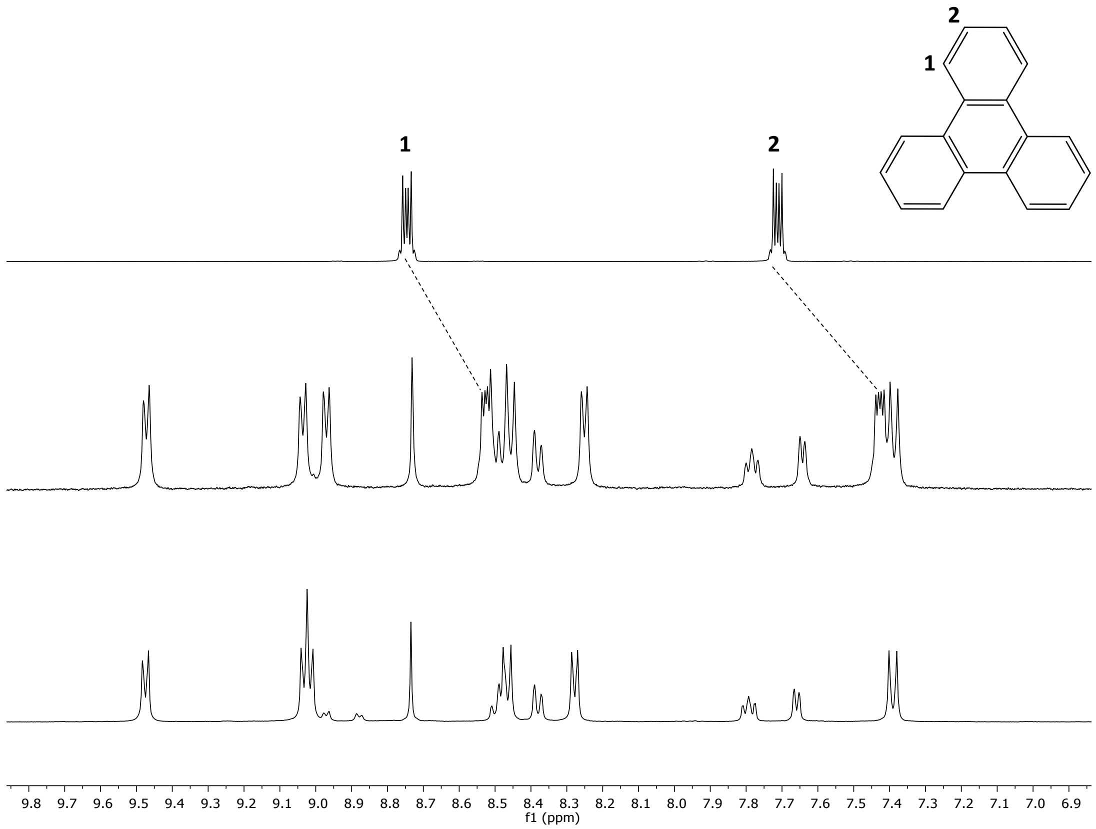

Figure S29. Aromatic region of the ^{1}H NMR spectra (500 MHz, CD_3CN, 298 K) of **4** (bottom), the prepared triphenylene ⊂ **4** host-guest complex (middle) and triphenylene (top)

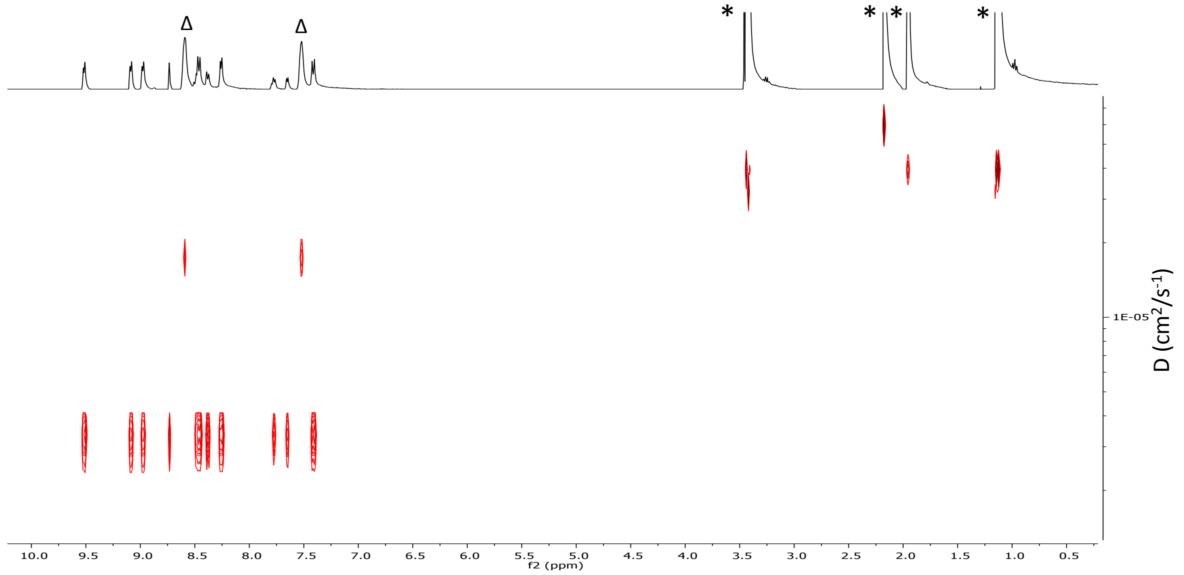

Figure S30. ^{1}H DOSY spectrum (400 MHz, CD_3CN, 298 K) of triphenylene ⊂ **4** host-guest complex. The diffusion coefficients for the host and the guest species in CD_3CN were measured to be 3.42 x 10^{-6} cm^2 s^{-1} and 2.61 x 10^{-5} cm^2 s^{-1} respectively. Δ indicate signals for protons of guest and * indicate signals for protons of residual solvents.

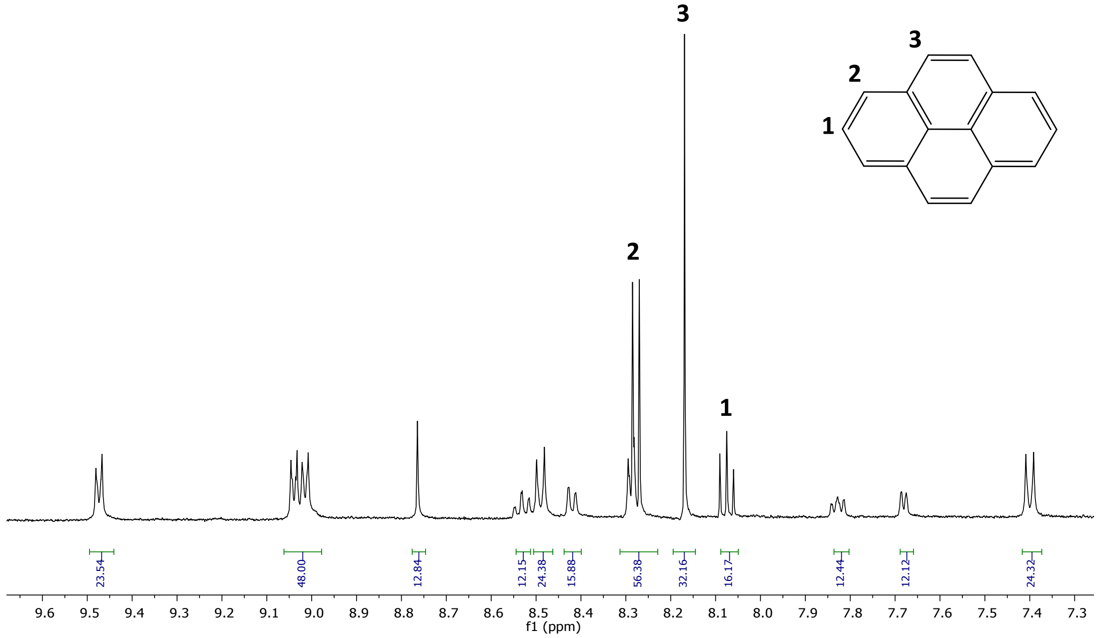

Figure S31. Aromatic region of the ^{1}H NMR spectrum (500 MHz, CD_3CN, 298 K) of the prepared pyrene ⊂ **4** host-guest complex.

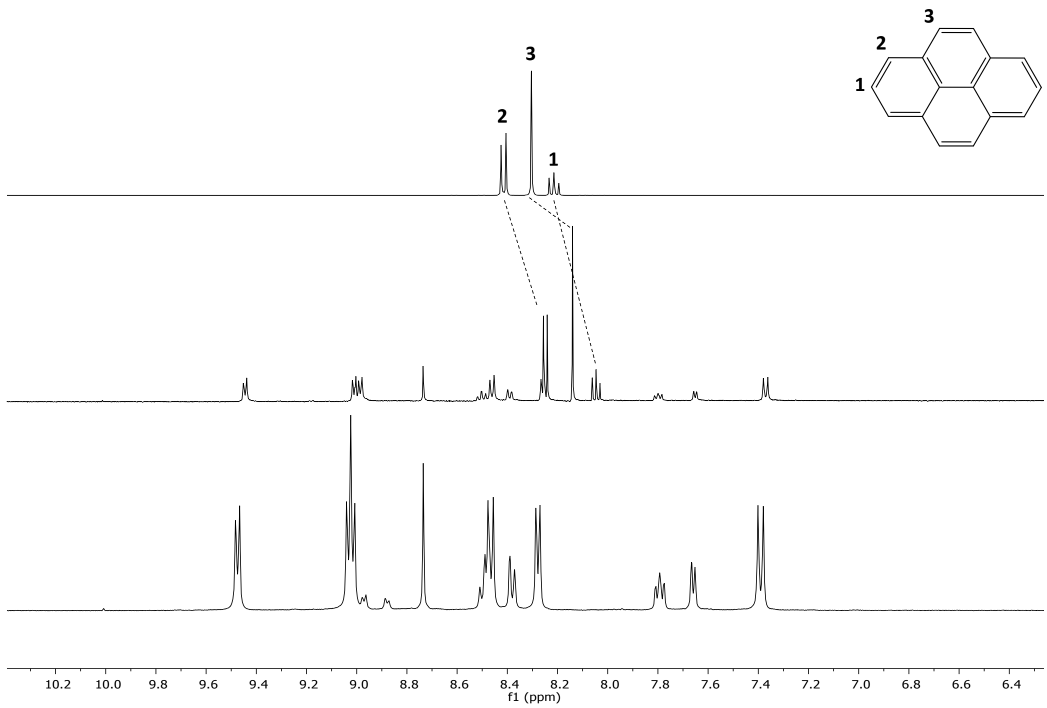

Figure S32. Aromatic region of the ^{1}H NMR spectra (500 MHz, CD_3CN, 298 K) of **4** (bottom), the prepared pyrene ⊂ **4** host-guest complex (middle) and pyrene (top)

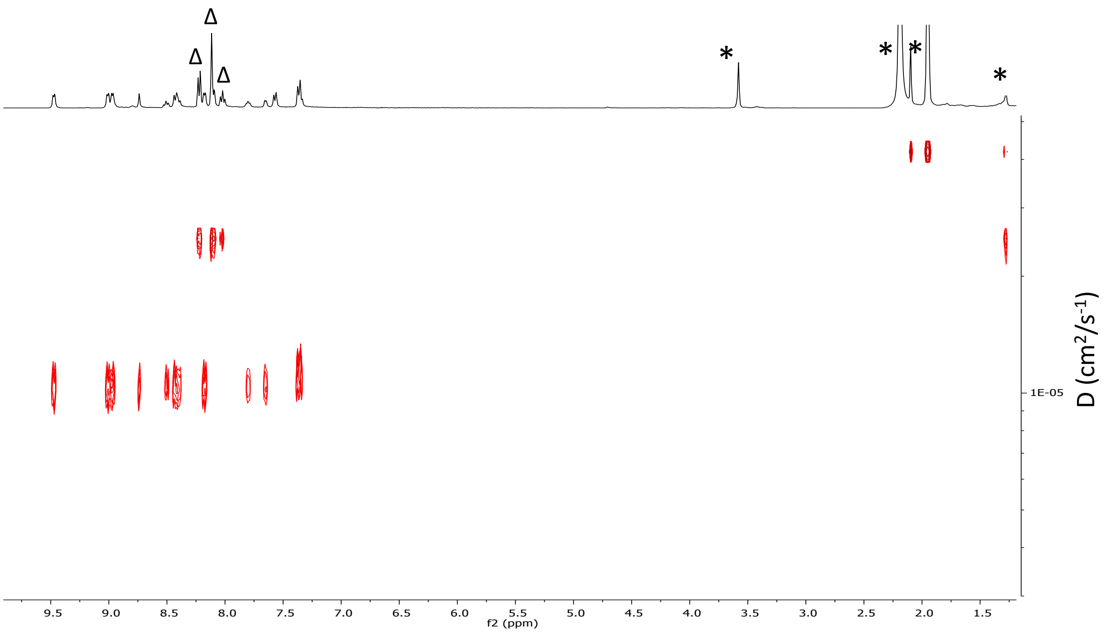

Figure S33. ^{1}H DOSY spectrum (400 MHz, CD_3CN, 298 K) of pyrene ⊂ **4** host-guest complex. The diffusion coefficients for the host and the guest species in CD_3CN were measured to be 3.43 x 10^{-6} cm^2 s^{-1} and 2.47 x 10^{-5} cm^2 s^{-1} respectively. Δ indicate signals for protons of guest and * indicate signals for protons of residual solvents.

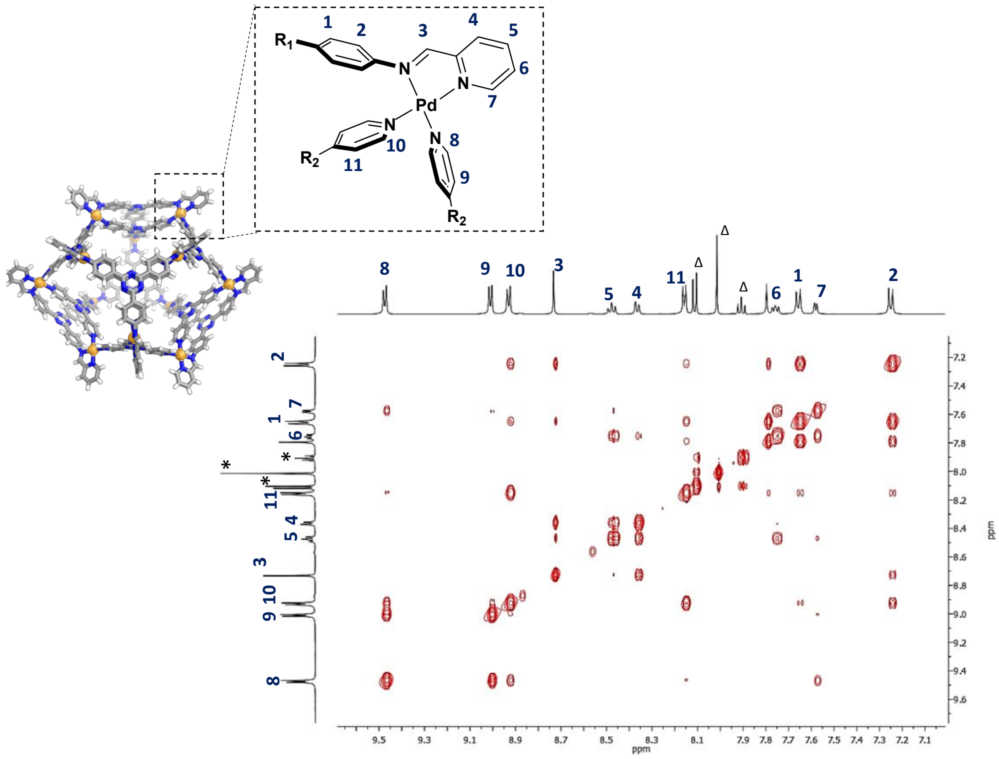

Figure S34. Aromatic region of ^{1}H-^{1}H NOESY spectrum (500 MHz, CD_3CN, 298 K) of pyrene ⊂ **4** host-guest complex, allowing identification of the signals for the triazine residues (H_8, H_9, H_{10} and H_{11}). Δ indicate signals for protons of guest

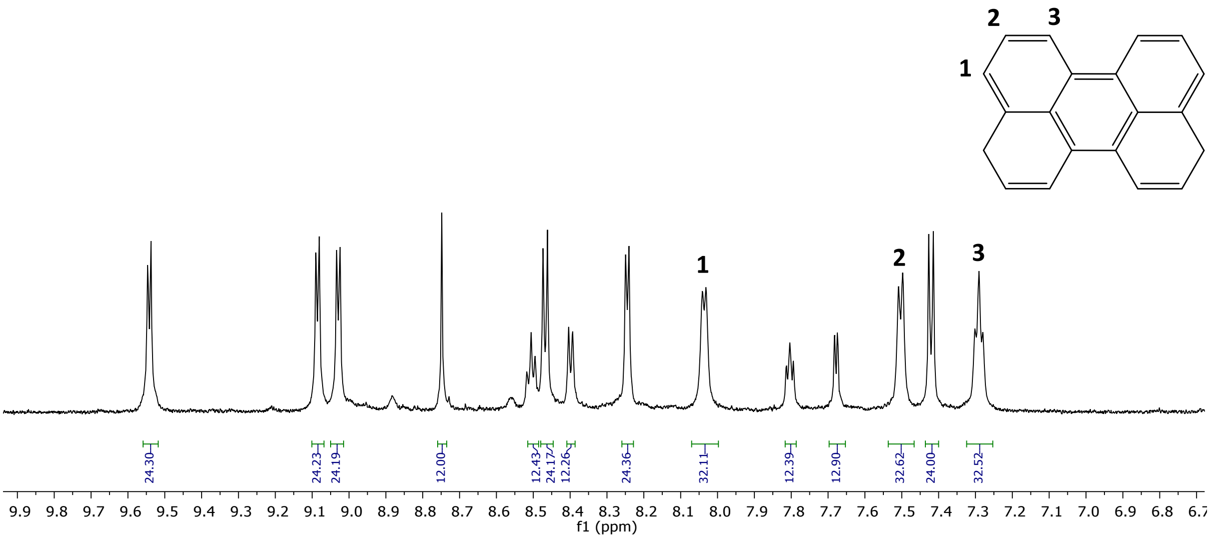

Figure S35. Aromatic region of the ^{1}H NMR spectrum (500 MHz, CD_3CN, 298 K) of the prepared perylene ⊂ **4** host-guest complex

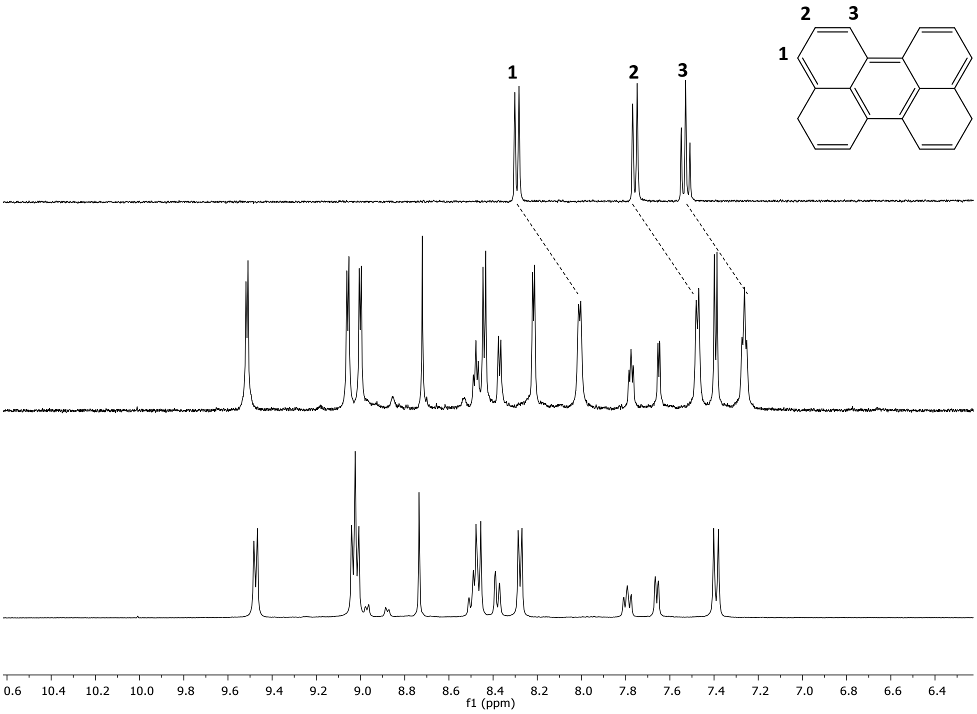

Figure S36. Aromatic region of the ^{1}H NMR spectra (500 MHz, CD_3CN, 298 K) of **4** (bottom), the prepared perylene ⊂ **4** host-guest complex (middle) and perylene (top)

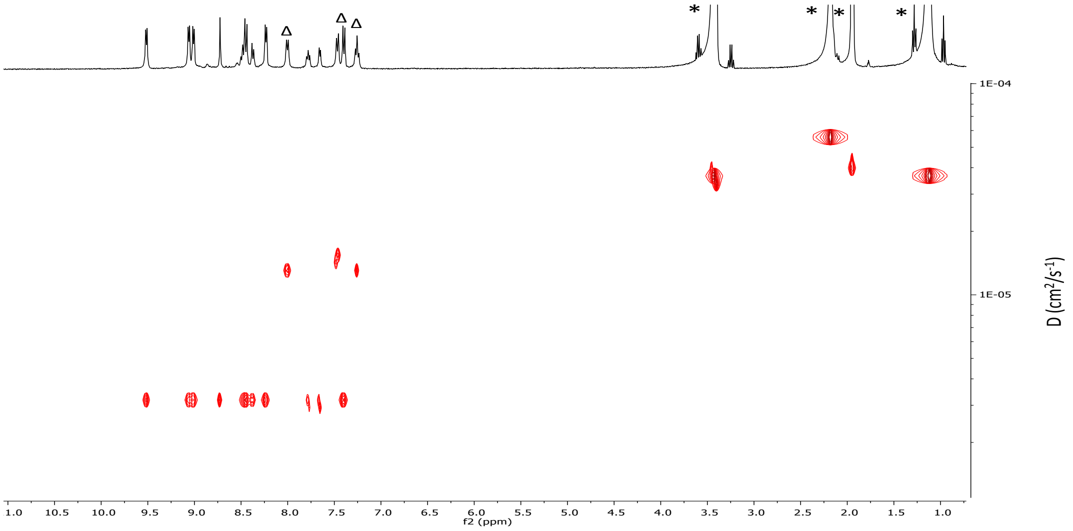

Figure S37. ^{1}H DOSY spectrum (400 MHz, CD_3CN, 298 K) of perylene ⊂ **4** host-guest complex. The diffusion coefficients for the host and the guest species in CD_3CN were measured to be 3.41 x 10^{-6} cm^2 s^{-1} and 2.52 x 10^{-5} cm^2 s^{-1} respectively. Δ indicate signals for protons of guest and * indicate signals for protons of residual solvents.

2.2 Determination of the association ratio and binding constant for the pyrene ⊂4 host-guest complex

Job-Plot, stoichiometry plot and Hill function titrations were carried out to determine the association ratio and binding constant. We observed the protons within the cavity of **4** to shift continuously upon the addition of guest, indicating that the shielding/de-shielding effect of the guest molecules on the cage increases gradually with the addition of guests. After addition of 90 eq. of guest, the signals of **4** remain basically unchanged upon the addition of further equivalents of guest. Meanwhile, the signals of the guest continued to shift toward lower field and approached those for the free guest molecule, indicating that the host-guest exchange rate becomes even faster after the cage is saturated. The Hill coefficient (n) for the pyrene ⊂ **4** system is calculated to be 1.02, indicating that the accommodation of pyrene by the cage is a non-cooperative process, which is in line with the independent interaction of each pyrene unit with one of the eight triazine residues present in the cage cavity .

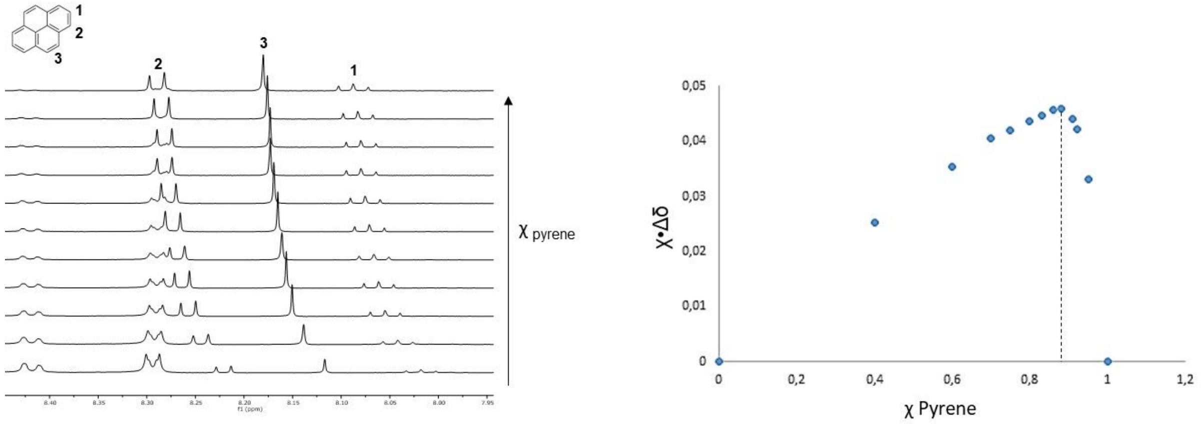

Figure S38. Partial ^1H NMR spectra (500 MHz, CD_3CN, 298 K) of the titration of **4** with pyrene, showing the chemical shifts of the pyrene shifting toward lower field upon increasing molar fraction of guest in solution (right). A Job plot revealed a 1:8 H:G ratio for the pyrene ⊂ **4** system (left). To carry out this experiment, a solution of **4** (100 µM) and a solution of pyrene (100 µM) were prepared and used as stock solutions. Different samples were prepared using different volumes of the stock solutions, changing the concentration of the guest continuously in the range of 0.00-100 µM, while the total concentration of **4** and pyrene remained constant along the experiment (100 µM). ^1H NMR spectra were collected after the host-guest adduct reached equilibrium (5 minutes).

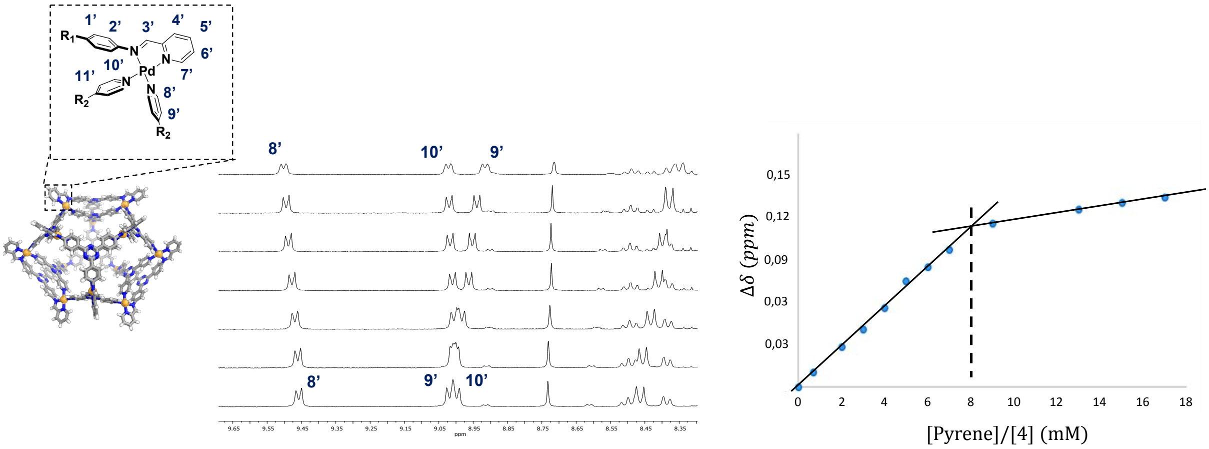

Figure S39. Partial ^1H NMR spectra (500 MHz, CD_3CN, 298 K) of the titration of **4** with pyrene, showing the chemical shifts of the host shifting toward higher field upon increasing molar fraction of guest in solution (right). ^1H-NMR stoichiometry plot for the titration of the titration of **4** with pyrene. The plot refers to the chemical shift of H_9 of **4**. A series of 4 mM solutions of **4** containing varying amounts of pyrene, ranging from 2.06 mM to 66 mM, was prepared. The solutions were permitted to equilibrate before they were examined by ^1H-NMR spectroscopy. The stoichiometry was found to be eight guest molecules per one host molecule using the mole ratio method[1] (left).

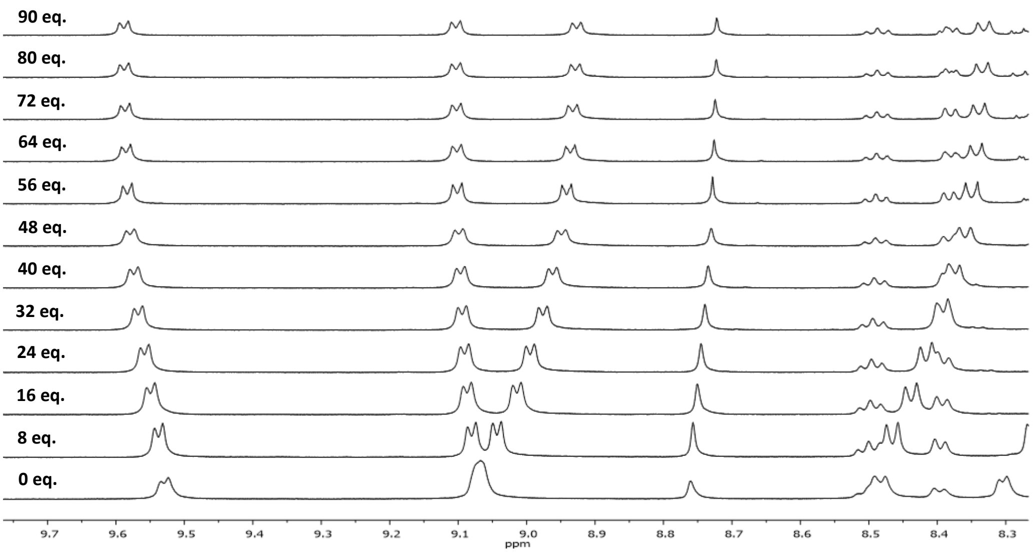

Figure S40. Partial ^{1}H NMR spectra (500 MHz, CD_3CN, 298 K) for the titration of pyrene into a solution of **4** ($c_{initial}$ = 80 µM). Equivalents of pyrene are with respect to **4**, and were determined using the integrals of the ^{1}H NMR signals for pyrene with respect to the signals for the internal standard (1,3,5-trimethoxybenzene). The concentration of the host was maintained at a constant value during the titration experiment.

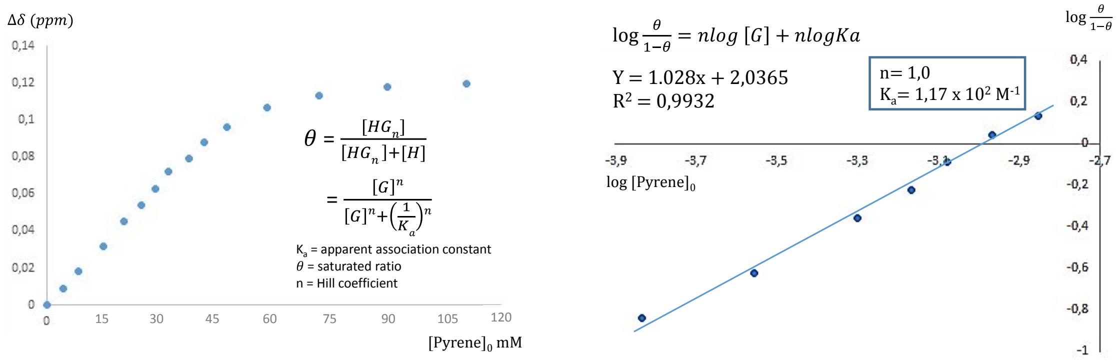

Figure S41. Titration curves of **4** and pyrene fitted with the Hill function**.** Δδ vs $[\text{Pyrene}]_0$ (right) and θ vs $\log[\text{pyrene}]_0$ (left). No evidence for either positive or negative cooperativity was revealed, with a Hill coefficient (n) of 1.028, characteristic of a non-cooperative binding mode.

2.3 Competitive encapsulation experiments

To a 160 µM solution of **4**, phenanthrene, 9,10-dimethyl anthracene, triphenylene, pyrene and perylene (10 equiv. each) were added and the mixture stirred at 80ºC for 2 h. After removal of the excess guests by filtration, the resulting solution was concentrated using a stream of N_2 and Et_2O (ca. 15 mL) was added. The yellow precipitate was dissolved with the minimum quantity of CD_3CN to prevent dissolving residual free guests remaining. The solution was filtrated and the ^1H NMR spectrum was measured to reveal preferential formation of pyrene ⊂ **4** (66%) and perylene ⊂ **4** (33%).

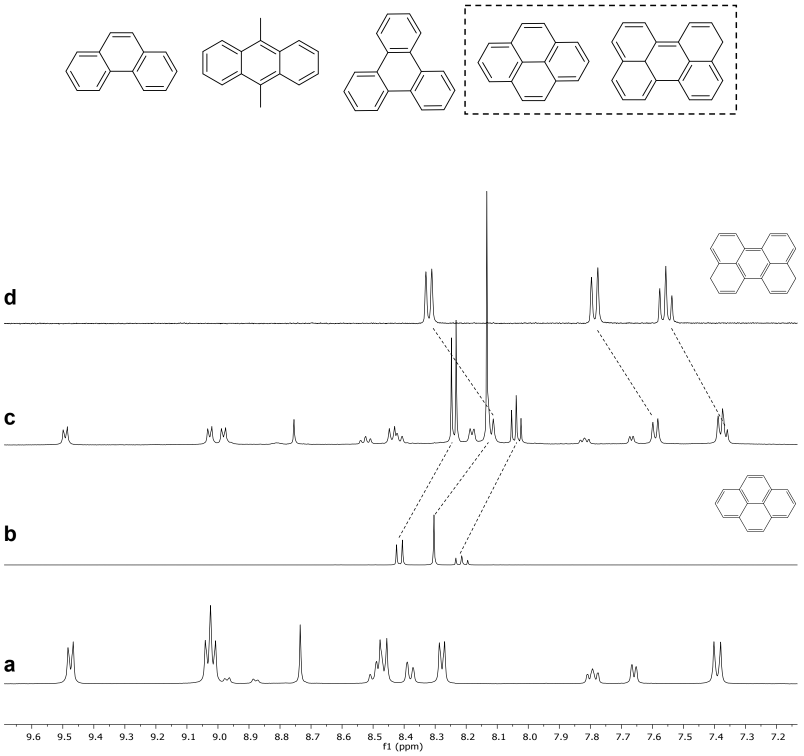

Figure S42. Aromatic region of the ^1H NMR spectra (500 MHz, CD_3CN, 298 K) of **4** (a), pyrene (b), host-guest complexes generated in the competitive binding experiment (c) and perylene (d)

To a 160 µM solution of **4**, phenanthrene, 9,10-dimethyl anthracene, triphenylene and perylene (10 equiv. each) were added and the mixture stirred at 80ºC for 2 h. After removal of the excess guests by filtration, the resulting solution was concentrated using a stream of N_2 and Et_2O (ca. 15 mL) was added. The yellow precipitate was dissolved with the minimum quantity of CD_3CN to prevent dissolving residual free guests remaining. The solution was filtrated and the ^1H NMR spectrum was measured to reveal preferential formation of perylene ⊂ **4** (75%) and triphenylene ⊂ **4** (25%).

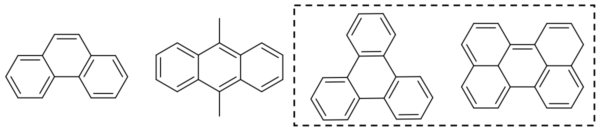

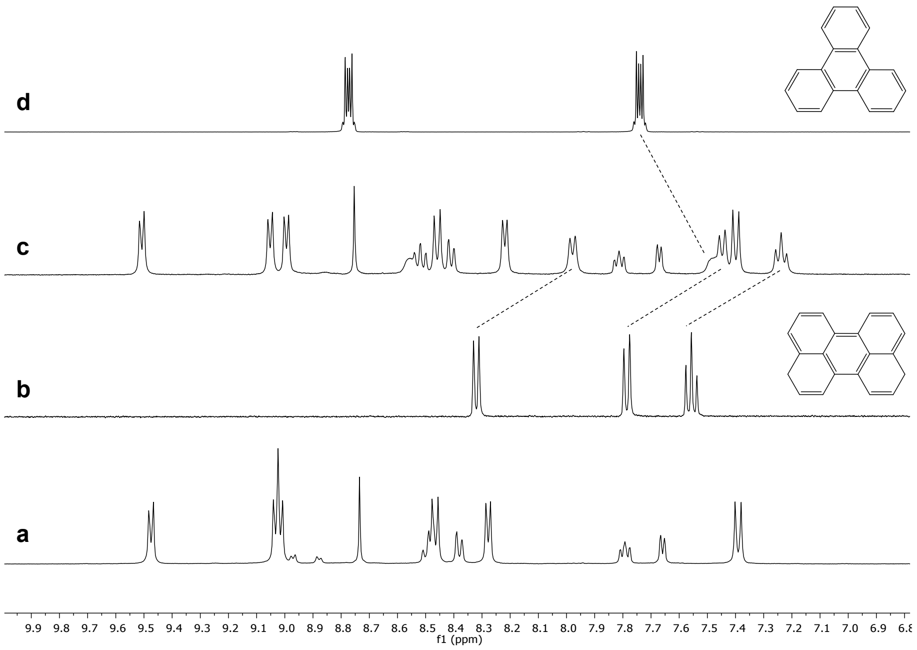

Figure S43. Aromatic region of the ^{1}H NMR spectra (500 MHz, CD_3CN, 298 K) of **4** (a), perylene (b), host-guest complexes generated in the competitive binding experiment (c) and triphenylene (d)

To a 160 µM solution of **4**, phenanthrene, 9,10-dimethyl anthracene and triphenylene, (10 equiv. each) were added and the mixture stirred at 80ºC for 2 h. After removal of the excess guests by filtration or centrifugation, the resulting solution was concentrated using a stream of N_2 and Et_2O (ca. 15 mL) was added. The yellow precipitate was dissolved with the minimum quantity of CD_3CN to prevent dissolving residual free guests remaining. The obtained solution was filtrated and the ^{1}H NMR spectrum was measured to reveal preferential formation of triphenylene ⊂ **4** (55%). However in this case the host guest complexes of all the guests present in the initial mixture were detected, indicating similar binding affinity to **4**.

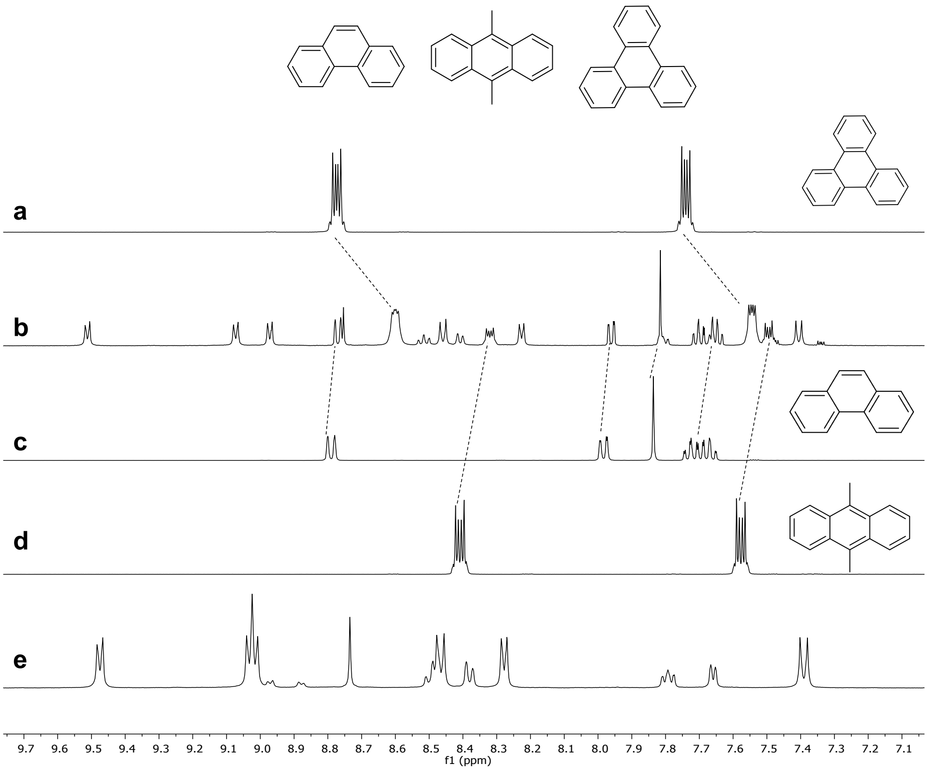

Figure S44. Aromatic region of the ^1H NMR spectra (500 MHz, CD_3CN, 298 K) of triphenylene (a), host-guest complexes generated in the competitive binding experiment (b), phenanthrene (c) 9,10-dimethyl anthracene (d) and **4** (e).

3 X-ray crystallography

Data were collected at Beamline I19 of Diamond Light Source employing silicon double crystal monochromated synchrotron radiation (0.6889 Å) with ω and *ψ* scans at 100(2) K.[1] Data integration and reduction were undertaken with Xia2.[2-4] Subsequent computations were carried out using the WinGX-32 graphical user interface.[5] Multi-scan empirical absorption corrections were applied to the data using the AIMLESS[6] tool in the CCP4 suite.[7] The structures were solved by direct methods using SHELXT[8] then refined and extended with SHELXL.[9] In general, non-hydrogen atoms with occupancies greater than 0.5 were refined anisotropically. Carbon-bound hydrogen atoms were included in idealised positions and refined using a riding model. Disorder was modelled using standard crystallographic methods including constraints, restraints and rigid bodies where necessary. Crystallographic data along with specific details pertaining to the refinement follow. Crystallographic data have been deposited with the CCDC (2175722-2175723).

[3]·6BF$_4$·3.75CH$_3$CN [+ solvent]

Formula $C_{70.50}H_{59.25}B_6F_{24}N_{21.75}Pd_3$, *M* 2051.20, Monoclinic, space group P 21/n (#14), *a* 18.39240(15), *b* 13.8060(2), *c* 33.9049(3) Å, *β* 97.4610(8), *V* 8536.43(17) Å^3, D_c 1.596 g cm^{-3}, *Z* 4, crystal size 0.10 by 0.05 by 0.02 mm, colour pale yellow, habit block, temperature 100(2) Kelvin, (Synchrotron) 0.6889 Å, (Synchrotron) 0.665 mm^{-1}, $T(\text{Analytical})_{min,max}$ 0.8114742034448892, 1.0, $2\vartheta_{max}$ 64.00, *hkl* range -28 28, -21 21, -52 52, *N* 164565, N_{ind} 32469(R_{merge} 0.0374), N_{obs} 18891(I > 2(I)), N_{var} 1380, residuals* $R1(F)$ 0.0620, $wR2(F^2)$ 0.2010, GoF(all) 1.031, $_{min,max}$ -2.649, 2.910 e$^-$ Å^{-3}.

*$R1$ = ||F_o| - |F_c||/|F_o| for F_o > 2(F_o); $wR2$ = (w(F_o^2 - F_c^2)^2/(wF_c^2)^2)$^{1/2}$ all reflections

w=1/[^{2}(F_o^2)+(0.1200P)^2] where P=(F_o^2+2F_c^2)/3

Specific refinement details:

The crystals of [**3**]·6BF$_4$·3.75CH$_3$CN [+ solvent] were grown by diffusion of ethyl acetate into an acetonitrile solution of the complex. Rapid handling prior to flash cooling in liquid nitrogen and the use of synchrotron radiation enabled high resolution data to be collected. The asymmetric unit was found to contain one complete assembly and associated counterions and solvent molecules. The anions within the structure show evidence of substantial disorder. Five of the tetrafluoroborate anions were modelled as disordered over two or three locations and one lattice site was modelled as disordered mixture of acetonitrile and tetrafluoroborate. Substantial bond length and thermal parameter restraints were applied to facilitate a reasonable refinement of the disordered anions and the lowest occupancy anions were modelled with isotropic thermal parameters. One coordinated acetonitrile was also modelled as disordered over two locations.

A remaining area of diffuse electron density corresponding to highly disordered solvent could not be modelled despite many attempts using restraints or rigid bodies. Consequently the SQUEEZE[10] function of PLATON[11] was employed to remove the contribution of the electron density associated with this highly disordered solvent, which gave a potential solvent accessible void of 299 Å^3 per unit cell (a total of approximately 90 electrons). The diffuse solvent molecule could not be conclusively assigned to acetonitrile or ethyl acetate and was therefore not included in the formula.

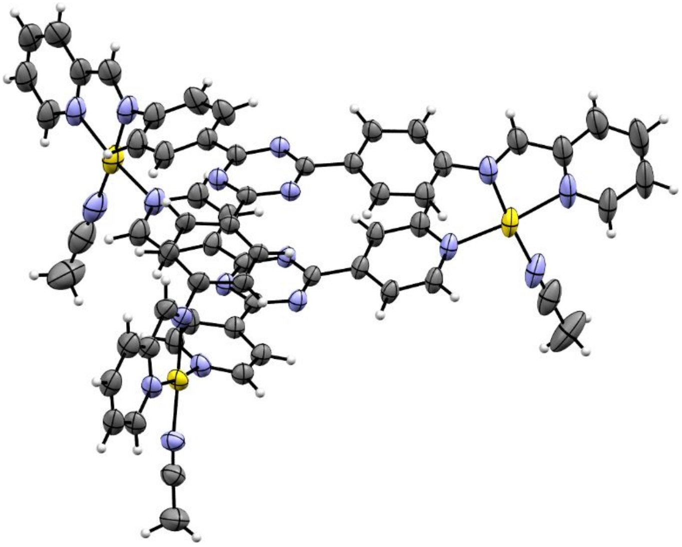

Figure S45. Ortep-style plot of the cationic portion of the crystal structure of [**3**]·$6BF_4$·$3.75CH_3CN$ [+ solvent], showing thermal ellipsoids at a probability of 50%.

[4]·$24NTf_2$ [+ solvent]

Formula $C_{348}H_{204}F_{144}N_{108}O_{96}Pd_{12}S_{48}$, *M* 12985.86, Tetragonal, space group I 41/a c d (#142), *a* 44.2321(2), *b* 44.2321(2), *c* 72.3147(10) Å, *V* 141482(2) Å^3, D_c 1.219 g cm^{-3}, *Z* 8, crystal size 0.100 by 0.060 by 0.050 mm, colour pale yellow, habit block, temperature 100(2) Kelvin, □(Synchrotron) 0.6889 Å, □(Synchrotron) 0.485 mm^{-1}, T(Analytical)$_{min,max}$ 0.9055573652334695, 1.0, 2□$_{max}$ 36.49, *hkl* range -40 38, -40 40, -65 65, *N* 179866, N_{ind} 13892(R_{merge} 0.0442), N_{obs} 5503(I > 2□(I)), N_{var} 1101, residuals* $R1(F)$ 0.0879, $wR2(F^2)$ 0.2870, GoF(all) 0.852, □□$_{min,max}$ -0.339, 0.420 e$^-$ Å^{-3}.

*$R1$ = □||F_o| - |F_c||/□|F_o| for F_o > 2□(F_o); $wR2$ = (□w(F_o^2 - F_c^2)^2/□(wF_c^2)^2)$^{1/2}$ all reflections

w=1/[□^{2}(F_o^2)+(0.1977P)^2] where P=(F_o^2+2F_c^2)/3

Specific refinement details:

The crystals of [**4**]·24NTf$_2$ [+ solvent] were grown by diffusion of diispropyl ether into an acetonitrile solution of the complex. The crystals employed were very weakly diffracting and immediately lost solvent after removal from the mother liquor. Rapid handling prior to flash cooling in liquid nitrogen and the use of synchrotron radiation were required to collect data. Consequently few reflections at greater than 1.1 Å resolution were observed and the quality of the integration is less than ideal. Nevertheless, the quality of the data is far more than sufficient to establish the connectivity of the structure. The asymmetric unit was found to contain one quarter of a $Pd_{12}L_4L_8'$ assembly and associated counterions.

Due to the limited resolution of the data and high level of thermal motion (or minor unresolved disorder) present throughout the structure, the GRADE program[12] was employed, using the GRADE Web Server,[13] to generate a full set of bond distance and angle restraints (DFIX, DANG, FLAT) for each of the organic ligands. Thermal parameter restraints (SIMU, RIGU) were applied to all atoms except for palladium to facilitate anisotropic refinement.

The anions within the structure also show evidence of thermal motion or disorder which could not be resolved and substantial bond length and angle restraints were required to achieve a reasonable model. The occupancies of the anions were initially refined and then fixed at the obtained values. Further reflecting the solvent loss and poor diffraction properties there is a substantial amount of void volume in the lattice containing smeared electron density from disordered solvent and ca. 21 unresolved anions per $Pd_{12}L_4L_8'$ assembly (included as triflimide in the formula). These anions and were significantly disordered and despite numerous attempts at modelling, no satisfactory model for the electron-density associated with them could be found. Consequently the SQUEEZE[10] function of PLATON[11] was employed to remove the contribution of the electron density associated with these remaining anions and further highly disordered solvent, which gave a potential solvent accessible void of 89533 Å^3 per unit cell (a total of approximately 33403 electrons). Diffuse solvent molecules could not be assigned to acetonitrile or diisoproyl ether and were therefore not included in the formula. Consequently, the molecular weight and density given above are underestimated.

CheckCIF gives one A level alert resulting from the limited resolution of the data and one B level alert (Large Average Ueq) resulting from unresolved disorder of a triflimide anion which is located on a special position.

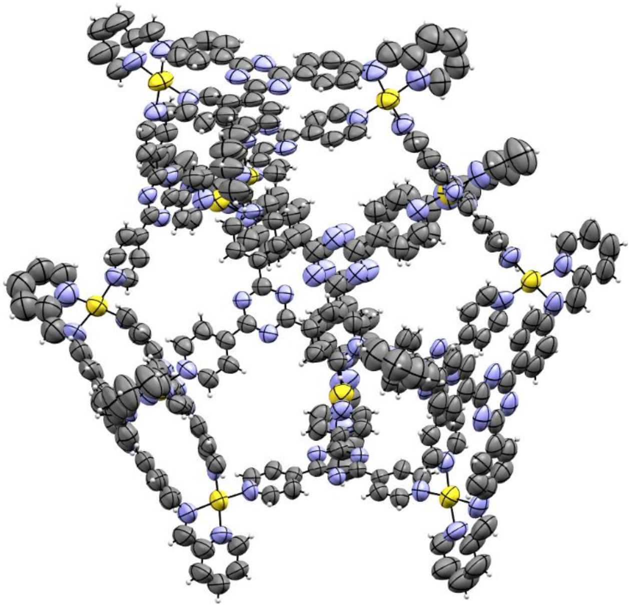

Figure S46. Ortep-style plot of the cationic portion of the crystal structure of [**4**]·24NTf$_2$ [+ solvent], showing thermal ellipsoids at a probability of 40%. The asymmetric unit contains one quarter of a **4** assembly.

4 Volume calculations

To determine the internal void space within the host **4**, Molovol[14] has been used to carry out the calculations based on the crystal structure. The standard parameters used for the calculation are:

Probe mode: one probe

Probe radius: 2.2 Å Grid resolution: 0.1 Å

Optimization depth: 4

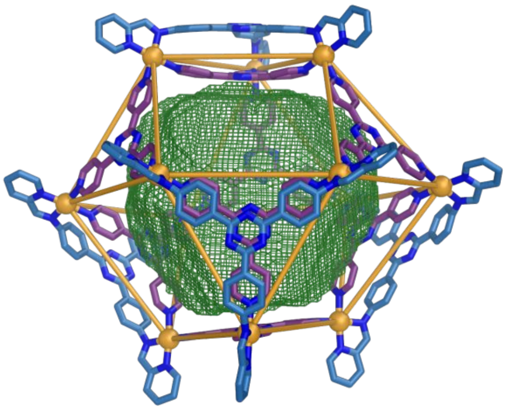

Figure S47. The Molovol calculated void space (green surface) within the crystal structure of **4** is 2631 Å^3.

5 Molecular Modelling

Geometry optimized host-guest structure were modelled at the PM6 level of theory using the program SCIGRESS. Molecular modelling was carried out by docking 8 pyrene guest units in the X-ray structure of **4** and minimizing using MMFF, with the following parameters: RHF (restricted HartreeFock Hamiltonian), LBFGS (low memory Broyden-Fletcher-Goldfarb-Shanno procedure), a maximum of 2000 SCF (self-consistent field) iterations and a SCF criterion at 10^{-4} kcal/mol.

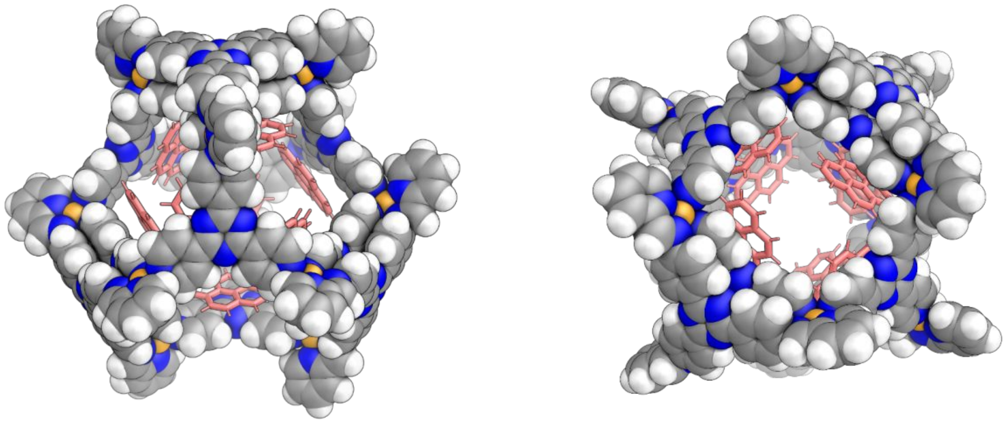

Figure S48. Molecular modelling of the 8:1 host-guest adduct consisting in eight pyrenes units within the cavity of the cuboctahedron **4**.

6 Temperature optimization of the conversion of 3 into 4

Three different solutions containing 0.5 mL of **3** (200 µM) in CD_3CN were prepared, using 1,3,5-trimethoxybenzene as an internal standard for the calibration of concentrations. The samples were heated to 25 °C, 40 °C and 60 °C in NMR tubes. After seven days, the samples were filtered to remove the solid generated and analyzed by ^{1}H NMR.

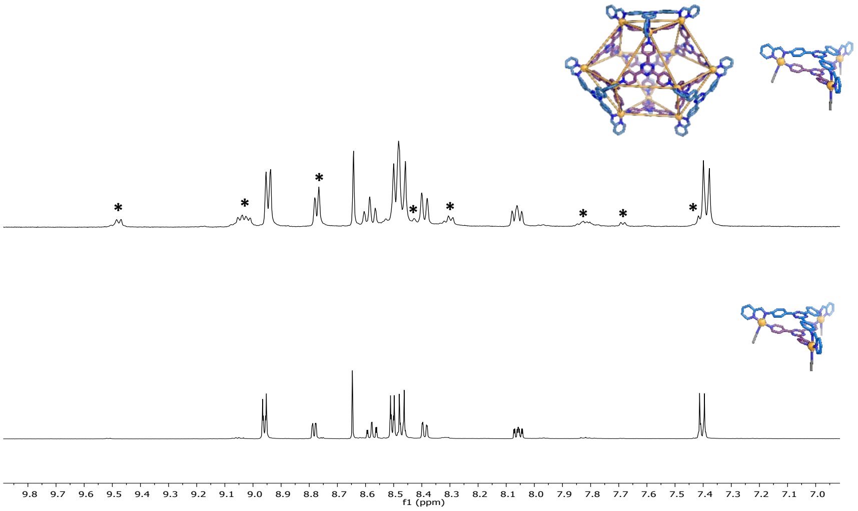

Figure S49. Partial ^{1}H NMR spectra (CD_3CN, 500 MHz, 298 K) monitoring the partial conversion of **3** (bottom) into **4** (top) at 25 °C after 7 days. * indicate signals corresponding to cuboctahedron **4**.

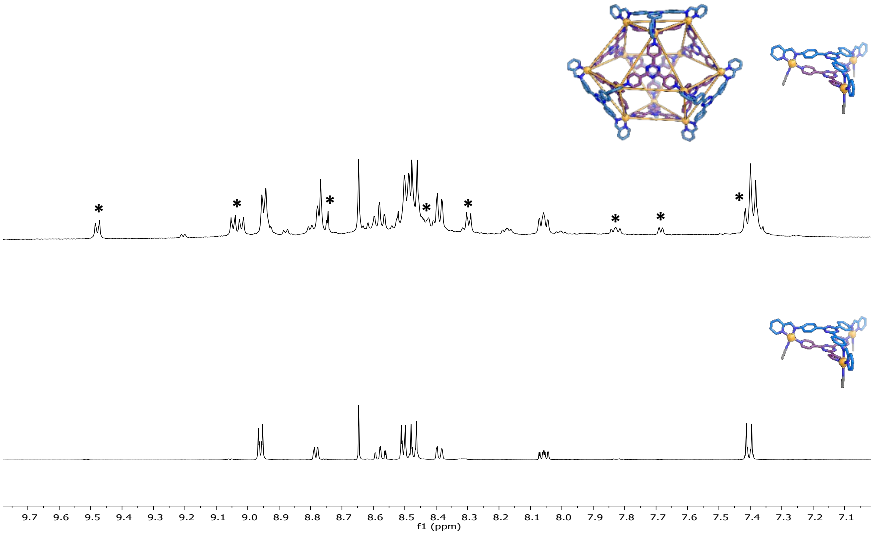

Figure S50. Partial ^{1}H NMR spectra (CD_3CN, 500 MHz, 298 K) monitoring the partial conversion of **3** (bottom) into **4** (top) at 40 °C after 7 days. * indicate signals corresponding to cuboctahedron **4**.

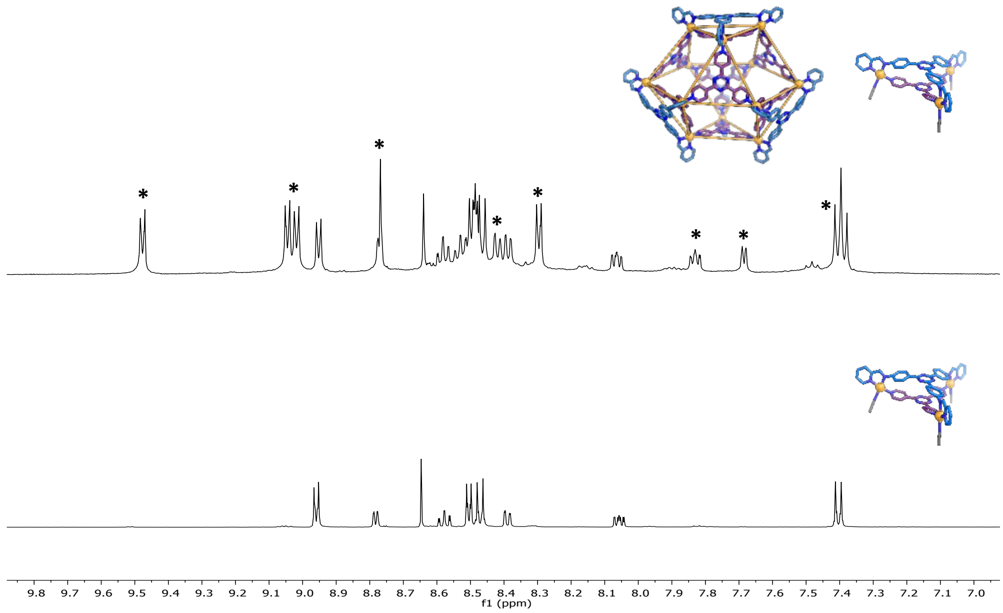

Figure S51. Partial ^{1}H NMR spectra (CD_3CN, 500 MHz, 298 K) monitoring the partial conversion of **3** (bottom) into **4** (top) at 60 °C after 7 days. * indicate signals corresponding to cuboctahedron **4**.

3 References

(1) Meyer, A. S.; Ayers, G. H. The Mole Ratio Method for Spectrophotometric Determination of Complexes in Solution. J. Am. Chem. Soc. 1957, 79, 49-53

(2) Allan, D.; Nowell, H.; Barnett, S.; Warren, M.; Wilcox, A.; Christensen, J.; Saunders, L.; Peach, A.; Hooper, M.; Zaja, L.; Patel, S.; Cahill, L.; Marshall, R.; Trimnell, S.; Foster, A.; Bates, T.; Lay, S.; Williams, M.; Hathaway, P.; Winter, G.; Gerstel, M.; Wooley, R. Crystals 2017, 7, 336.

(3) Evans, P. Acta Cryst. 2006, D62, 72.

(4) Winter, G. J. Appl. Crystallogr. 2010, 43, 186.

(5) Winter, G.; Waterman, D. G.; Parkhurst, J. M.; Brewster, A. S.; Gildea, R. J.; Gerstel, M.; Fuentes-Montero, L.; Vollmar, M.; Michels-Clark, T.; Young, I. D.; Sauter, N. K.; Evans, G. Acta Cryst. 2018, D74, 85.

(6) Farrugia, L. J. Appl. Crystallogr. 2012, 45, 849.

(7) Evans, P. R.; Murshudov, G. N. Acta Cryst. 2013, D69, 1204.

(8) Winn, M. D.; Ballard, C. C.; Cowtan, K. D.; Dodson, E. J.; Emsley, P.; Evans, P. R.; Keegan, R. M.; Krissinel, E. B.; Leslie, A. G. W.; McCoy, A.; McNicholas, S. J.; Murshudov, G. N.; Pannu, N. S.; Potterton, E. A.; Powell, H. R.; Read, R. J.; Vagin, A.; Wilson, K. S. Acta Cryst. 2011, D67, 235.

(9) Sheldrick, G. Acta. Cryst. 2015, A71, 3.

(10) Sheldrick, G. M. Acta. Cryst. 2015, C71, 3.

(11) van der Sluis, P.; Spek, A. L. Acta Cryst. 1990, A46, 194.

(12) Spek, A. L. PLATON: A Multipurpose Crystallographic Tool; Utrecht University: Utrecht, The Netherlands, 2008.

(13) Bricogne, G.; Blanc, E.; Brandle, M.; Flensburg, C.; Keller, P.; Paciorek, W.; Roversi, P.; Sharff, A.; Smart, O. S.; Vonrhein, C.; Womack, T. O. BUSTER; 2.11.2 ed.; Global Phasing Ltd.: Cambridge, United Kingdom, 2011.

(14) Smart, O. S.; Womack, T. O. Grade Web Server; Global Phasing Ltd., 2014.

(15) Maglic, J. B.; Lavendomme, R., An Easy-to-Use Program to Calculate Various Volumes and Surface Areas of Chemical Structures and Identify Cavities. doi.org/10.33774/chemrxiv-2021-dss1j
